# Ferritin H deficiency deteriorates cellular iron handling and worsens *Salmonella typhimurium* infection by triggering hyperinflammation

**DOI:** 10.1172/jci.insight.141760

**Published:** 2021-07-08

**Authors:** David Haschka, Piotr Tymoszuk, Verena Petzer, Richard Hilbe, Simon Heeke, Stefanie Dichtl, Sergej Skvortsov, Egon Demetz, Sylvia Berger, Markus Seifert, Anna-Maria Mitterstiller, Patrizia Moser, Dirk Bumann, Manfred Nairz, Igor Theurl, Guenter Weiss

**Affiliations:** 1 Department of Internal Medicine II, Medical University of Innsbruck, Innsbruck, Austria.; 2 Department of Therapeutic Radiology and Oncology, Laboratory for Experimental and Translational Research on Radiation Oncology, Tyrolean Cancer Research Institute, Medical University of Innsbruck, Innsbruck, Austria.; 3 Institute of Pathology, INNPATH, Innsbruck, Austria.; 4 Biozentrum, University of Basel, Klingelbergstrasse, Basel, Switzerland.

**Keywords:** Immunology, Infectious disease, Bacterial infections, Innate immunity

## Abstract

Iron is an essential nutrient for mammals as well as for pathogens. Inflammation-driven changes in systemic and cellular iron homeostasis are central for host-mediated antimicrobial strategies. Here, we studied the role of the iron storage protein ferritin H (FTH) for the control of infections with the intracellular pathogen *Salmonella enterica* serovar Typhimurium by macrophages. Mice lacking FTH in the myeloid lineage (*LysM-Cre^+/+^Fth^fl/fl^* mice) displayed impaired iron storage capacities in the tissue leukocyte compartment, increased levels of labile iron in macrophages, and an accelerated macrophage-mediated iron turnover. While under steady-state conditions, *LysM-Cre^+/+^Fth^+/+^* and *LysM-Cre^+/+^Fth^fl/fl^* animals showed comparable susceptibility to *Salmonella* infection, i.v. iron supplementation drastically shortened survival of *LysM-Cre^+/+^Fth^fl/fl^* mice. Mechanistically, these animals displayed increased bacterial burden, which contributed to uncontrolled triggering of NF-κB and inflammasome signaling and development of cytokine storm and death. Importantly, pharmacologic inhibition of the inflammasome and IL-1β pathways reduced cytokine levels and mortality and partly restored infection control in iron-treated ferritin-deficient mice. These findings uncover incompletely characterized roles of ferritin and cellular iron turnover in myeloid cells in controlling bacterial spread and for modulating NF-κB and inflammasome-mediated cytokine activation, which may be of vital importance in iron-overloaded individuals suffering from severe infections and sepsis.

## Introduction

Iron is essential for numerous metabolic processes in living cells, including heme production, oxidative phosphorylation, and DNA synthesis, and it also acts as a cofactor of multiple metalloenzymes (1). Nevertheless, a surplus of iron can be potentially harmful, for instance, as a catalyst of nonenzymatic generation of toxic oxygen radicals via Fenton/Haber–Weiss chemistry (2). In addition, iron is a critical nutrient for growth and virulence of most bacterial pathogens (3). Thus, during infection, the availability of iron is limited by the host and, as a result, the replication of invading pathogens is restricted — a process known as “nutritional immunity” (4). Knowledge about the mechanisms of iron withholding has emerged in the last decades, pointing to a dependency on the localization and nature of a pathogen (5–7). Infections with intracellular bacteria such as *Salmonella* or *Listeria* lead to increased iron efflux from bacteria-hosting macrophages to limit intracellular iron availability (8, 9). In contrast, infection with extracellular bacteria promotes scavenging of extracellular iron by macrophages, resulting in systemic hypoferremia and macrophage iron accumulation (10).

*Salmonella* is a Gram-negative pathogen, which is mainly localized in a modified late phagosome known as the *Salmonella*-containing vacuole (SCV) of macrophages (11). *Salmonella* pathogenicity relies on its ability to invade macrophages and to use those cells as a habitat for multiplication and spreading within the host (12). The intracellular replication of this pathogen is highly dependent on iron availability in the SCV (13–15).

Iron homeostasis within macrophages is regulated at multiple levels. Under steady state, macrophages take part in systemic iron homeostasis by taking up, degrading, and recycling old or damaged erythrocytes (16). As the degradation of heme produces significant amounts of iron, macrophages are perfectly equipped to handle this potentially toxic trace metal. One such mechanism is increased iron efflux, which is mediated by the sole known iron exporter (FPN1) (17). The other option is the storage of intracellular iron within ferritin. Ferritin is a protein polymer consisting of a total of 24 ferritin H (FTH) and ferritin L (FTL) subunits forming a round structure that can store up to 4500 Fe^3+^ ions (18). The stoichiometric proportion of the L and the H subunits differs, depending on the tissue. However, only the H subunit possesses ferroxidase activity that is necessary for oxidation of cytoplasmic ferrous iron and its incorporation into the ferritin oligomer but also affects dietary iron absorption (19). Moreover, macrophages produce FTH-rich molecules, as they are exposed to significant variation of labile iron levels (20), and can release ferritin by specific pathways (21).

Upon infection with intracellular bacteria, the cellular iron turnover in the macrophage is substantially altered to deprive invading pathogens of this essential nutrient (5, 6). In the case of *Salmonella* infection, macrophages upregulate FPN1 expression and, therefore, reduce the cytoplasmic labile iron pool (22). However, ferritin protein levels are also upregulated, although incorporation of iron is reduced (9). This is biologically reasonable, as *Salmonella* may be able to access ferritin-associated iron (23). Cellular regulation of ferritin and FPN1 translation is mediated by iron regulatory proteins (IRPs) 1 and 2, which respond to changes in cellular iron levels. IRP deletion in macrophages leads to hyperferritinemia, and this in turn seems to increase iron availability for invading intracellular pathogens like *Salmonella,* resulting in shortened survival of the KO animals (23).

Notably, protective effects of ferritin were described in diverse infection models, including infection with the intracellular, macrophage-colonizing pathogen *Mycobacterium tuberculosis* (24) and polymicrobial sepsis (25). In this setting, FTH expression and FTH-mediated control of cellular iron availability has been linked to disease tolerance and metabolic adaption (24). Recently, we demonstrated that iron loading of macrophages induces a pathogen-friendly metabolic environment by suppressing glycolysis and boosting Krebs cycle activity (26).

In light of these contrasting findings, we sought to clarify the effect of macrophage-expressed ferritin on immune response against the model intracellular pathogen *Salmonella enterica* serovar Typhimurium. To this end, we characterized the baseline iron turnover in *LysM-Cre^+/+^Fth^fl/fl^* mice lacking FTH in myeloid cells and delved into their susceptibility to *Salmonella* infection. Our results functionally link the increased labile cellular iron levels in FTH-deficient macrophages to rapidly expanding pathogen burdens, which contribute to uncontrolled activation of the NF-κB and inflammasome pathways, detrimental cytokine formation, and dramatically shortened survival in course of infection with the intramacrophage pathogen *S*. Typhimurium.

## Results

### FTH depletion increases cellular labile iron and iron efflux in macrophages.

FTH is the ferroxidase subunit of the ferritin complex, the activity of which is necessary for iron incorporation. Therefore, it is expected that *Fth* gene disruption severely impairs the iron storing capacity of cells and increases cellular levels of labile iron. To corroborate this assumption for FTH-deficient macrophages, we characterized alterations in iron turnover in mice lacking the *Fth* gene in the myeloid lineage (*LysM-Cre^+/+^Fth^fl/fl^* mice, herein referred to as *Fth*^Δ*/*Δ^ mice).

First, we could prove efficient deletion of the *Fth* gene in control and iron-treated bone marrow–derived macrophages (BMDMs) from *Fth*^Δ*/*Δ^ mice by immunoblotting. Notably, a compensatory upregulation of the other ferritin subunit, FTL, could be observed in *Fth*^Δ*/*Δ^ BMDMs as compared with *LysM-Cre^+/+^Fth^+/+^*-derived macrophages (herein referred to as *Fth^+/+^* macrophages) (Supplemental Figure 1; supplemental material available online with this article; https://doi.org/10.1172/jci.insight.141760DS1).

**Figure 1:**
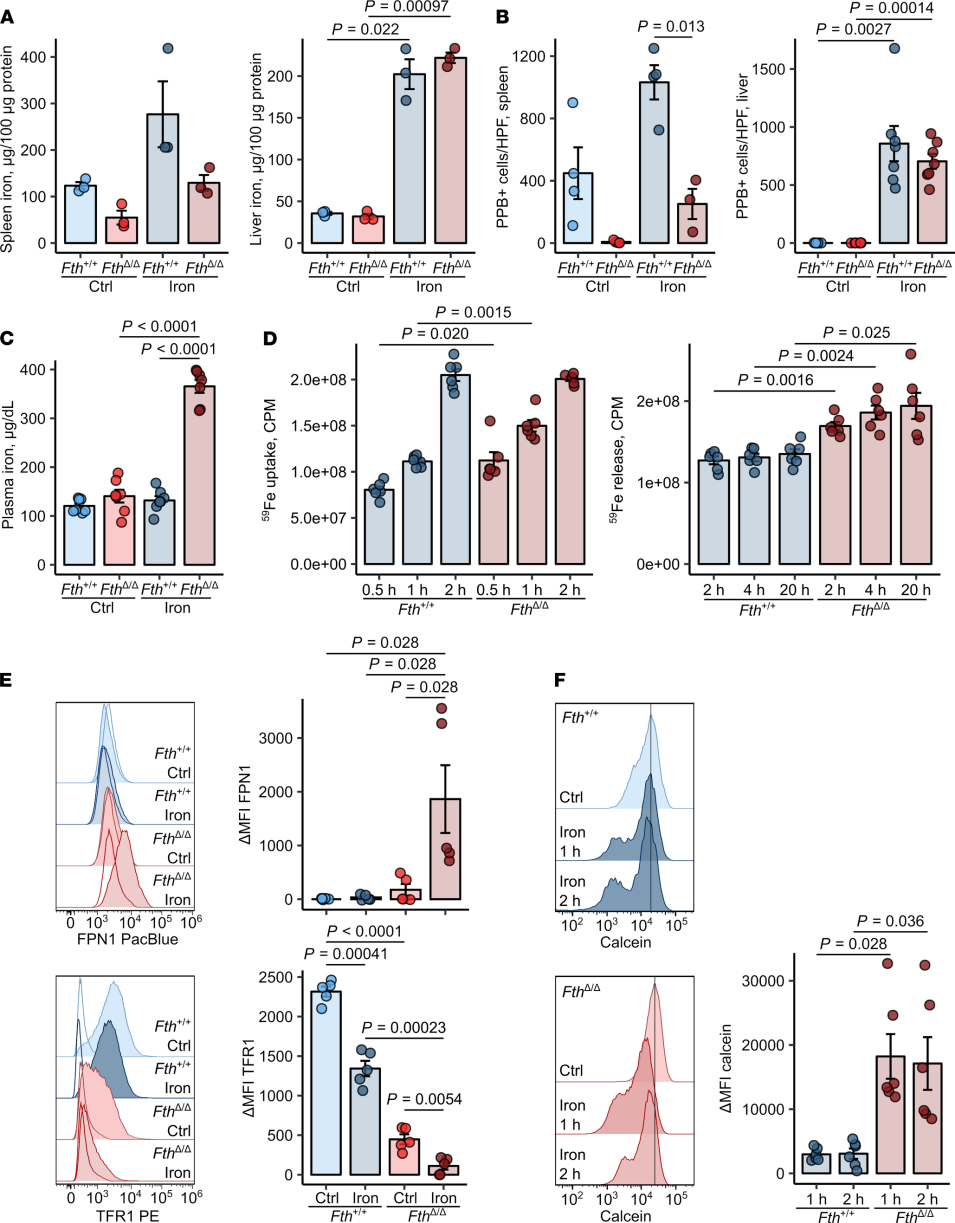
*Fth* deletion in myeloid cells increases cellular labile iron pool and iron turnover in macrophages. (**A–C**) *Fth^fl/fl^* (*Fth^+/+^)* and *LysM-Cre Fth^fl/fl^* (*Fth*^Δ*/*Δ^) mice were injected i.v. with iron isomaltoside (2 mg elementary Fe/mouse) or left untreated and analyzed 3 days later. (**A**) Nonheme iron content of the spleen and liver (*n* = 3 per group). (**B**) Counts of splenic and hepatic PPB-positive macrophages. Each point denotes mean from at least 3 high-power fields (HPFs) per animal (spleen: *Fth^+/+^* ctrl, *Fth^+/+^* iron, *Fth*^Δ*/*Δ^ ctrl — *n* = 4, *Fth*^Δ*/*Δ^ iron —*n* = 3; liver: *n* = 7 per group). (**C**) Nonheme iron concentrations in serum (*n* = 7 per group). (**D**) Iron uptake and release from *Fth^+/+^* and *Fth*^Δ*/*Δ^ bone marrow–derived macrophages (BMDMs). Iron uptake was measured in cultures stimulated with 5 μM ^59^Fe^3+^ (in form of FeCl_3_) for the indicated time points. Iron release from BMDMs loaded with 5 μM ^59^Fe^3+^ into culture supernatant was determined at the indicated time points (uptake and release: *n* = 6 per group). (**E**) Surface expression of FPN1 and TFR1 in *Fth^+/+^* and *Fth*^Δ*/*Δ^ BMDMs cultivated with/without 10 μM Fe^3+^ (FeCl_3_) for 12 hours was measured by flow cytometry (*n* = 5 per group). (**F**) Calcein-stained *Fth^+/+^* and *Fth*^Δ*/*Δ^ BMDMs were stimulated with 50 μM Fe^3+^ (FeCl_3_) for the indicated time points. Calcein fluorescence expressed as ΔMFI was determined by flow cytometry. Each point denotes single observation (**A** and **C**–**F**) or a mean from at least 3 HPFs per animal (**B**). Bars with whiskers represent mean ± SEM. Statistical significance was assessed with 2-way ANOVA (**A**–**C** and **E**), Kruskal-Wallis test (**E**, FPN1), or repeated-measures 2-way ANOVA (**E**, TFR1 data, and **F**) with Benjamini-Hochberg-corrected 2-tailed post hoc *t* tests (**A–F**) or Mann-Whitney *U* tests (**E**, FPN1). In the plots, post-hoc test *P* values are indicated.

Deletion of the *Fth* gene in the myeloid compartment led to a substantial decrease of splenic nonheme iron content and markedly reduced numbers of iron-storing Pearl’s Prussian Blue–positive resident splenic macrophages compared with those in *Fth^+/+^* spleens. These differences preexisted in steady state and persisted after challenge with i.v. iron isomaltoside, a clinically approved preparation that delivers iron-containing nanoparticles directly to macrophages (27, 28). However, no significant difference was observed in the liver iron content between *Fth*^Δ*/*Δ^ and *Fth^+/+^* mice (Figure 1, A and B). Under steady-state conditions, plasma iron levels were not affected by myeloid FTH deficiency, but these dramatically increased in *Fth*^Δ*/*Δ^ mice after iron challenge (Figure 1C). Interestingly, in this setting, serum hepcidin levels rose upon stimulation with iron, but were unaffected by lack of myeloid ferritin (Supplemental Figure 3A). Leukocyte composition in the liver and the spleen was also unaltered in iron-loaded *Fth*^Δ*/*Δ^ mice in comparison with that in *Fth^+/+^* mice, except for Ly6C^hi^ monocytes in the spleen, which were significantly increased in iron-loaded *Fth*^Δ*/*Δ^ mice (Supplemental Figure 3, B and C). Cumulatively, myeloid FTH deficiency seems to impair the iron storage capacity of the spleen myeloid cells and increases circulating iron levels.

To verify this on a cellular level, we investigated iron turnover rates in FTH-proficient and -deficient BMDMs. Interestingly, the iron uptake rate in *Fth*^Δ*/*Δ^ macrophages was initially slightly increased but leveled off 2 hours after iron challenge (Figure 1D). However, the iron efflux rates of FTH-deleted macrophages were significantly elevated as compared with WT cells (Figure 1D), suggesting reduced storage capacities in these cells. In line with the augmented export, total (Supplemental Figure 1) and surface levels (Figure 1E) of the sole iron exporter FPN1 were substantially increased in FTH-deficient cells at baseline and further boosted by iron challenge. Additionally, reduced iron storage capacities of FTH-deficient macrophages led to accumulation of cellular labile iron even without iron challenge, as measured by calcein quenching (Figure 1F). Expectedly, expression of the central iron uptake receptor TFR1 was reduced in FTH-deficient cells, a finding which is in line with the known negative effects of cellular labile iron accumulation on *Tfr1* mRNA stability (1).

Taken together, myeloid-specific *Fth*-KO mice have a defective cellular iron storage, which leads to an expansion of the cellular labile iron pool, FPN1 induction, and increased export exacerbating upon iron loading.

### Myeloid FTH deficiency causes hyperresponsiveness to Salmonella infection.

**Figure 2:**
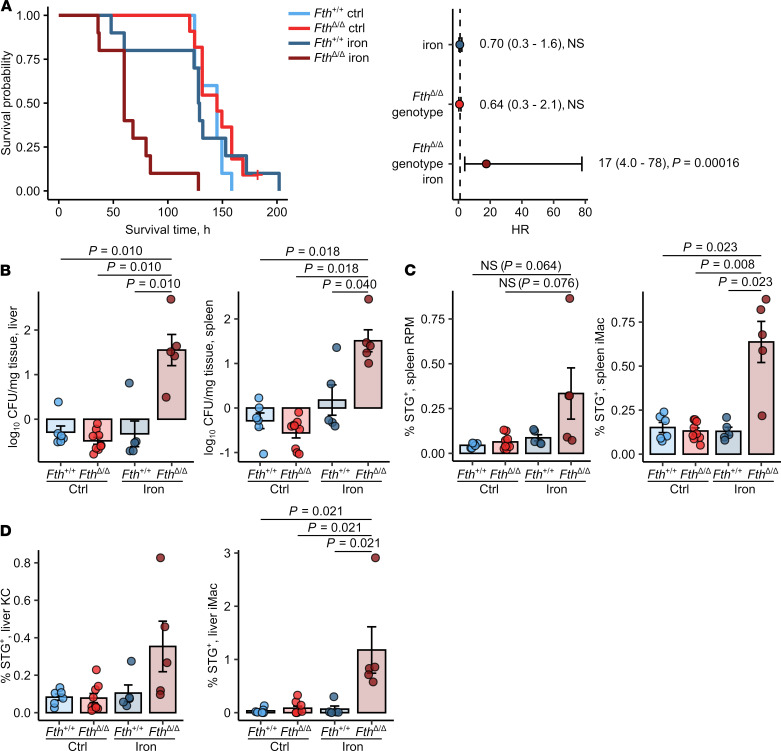
Loss of FTH in myeloid cells increases susceptibility of iron-loaded mice to *Salmonella* infection. *Fth^fl/fl^* (*Fth^+/+^*) and *LysM-Cre Fth^fl/fl^* (*Fth*^Δ*/*Δ^) mice were i.v. administered PBS or iron isomaltoside (2 mg elementary Fe per animal) and infected 3 days later with 500 CFU GFP-expressing *S*. Typhimurium (STG). (**A**) Surviving fractions of control and iron-loaded *Fth^+/+^* and *Fth*^Δ*/*Δ^ mice as a function of time after infection (*Fth^+/+^* ctrl, *Fth^+/+^* iron, *Fth*^Δ*/*Δ^ iron: *n* = 11, *Fth*^Δ*/*Δ^ ctrl: *n* = 10). The forest plots show results of Cox proportional hazard modeling of the data (HR, hazard ratio). (**B**) The number of GFP-expressing bacteria in the spleen and liver determined by flow cytometry of organ lysates 20 hours after infection (*Fth^+/+^* ctrl: *n* = 6, *Fth^+/+^* iron: *n* = 5, *Fth*^Δ*/*Δ^ ctrl: *n* = 9, *Fth*^Δ*/*Δ^ iron: *n* = 5). (**C** and **D**) Bacterial colonization of spleen red-pulp (RPM) and inflammatory macrophages (iMacs) (**C**) and of liver Kupffer cells (KCs) and iMac (**D**) 20 hours after was measured by flow cytometry and expressed as percent of STG-positive cells within the parent population (*Fth^+/+^* ctrl: *n* = 6, *Fth^+/+^* iron: *n* = 5, *Fth*^Δ*/*Δ^ ctrl: *n* = 9, *Fth*^Δ*/*Δ^ iron: *n* = 5). In **A**, data are presented as Kaplan-Meier plot and forest plot with points representing Cox regression estimates and whiskers depicting 95% CI for the estimates. In the other panels, each point denotes a single animal; bars with whiskers represent mean ± SEM. In **A**, statistical significance was assessed with Cox proportional hazard modeling for genotype, iron, and genotype: iron interaction terms, points in the forest plot are labeled with estimate values, 95% CI and *P* values. In other panels, statistical significance was assessed with Kruskal-Wallis test and with Benjamini-Hochberg-corrected Mann-Whitney *U* tests. In the plots, post hoc test *P* values are indicated.

Next, we determined how myeloid FTH KO affects susceptibility to systemic infection with the intracellular bacterium, *S*. Typhimurium. Upon i.p. infection, we could not find a survival difference between control-treated *Fth^+/+^* and *Fth*^Δ*/*Δ^ mice. In turn, i.v. iron loading prior to infection dramatically shortened the survival of *Fth*^Δ*/*Δ^ mice as compared with *Fth^+/+^* mice (median survival 60 hours vs. 128.5 hours for iron-loaded *Fth^+/+^*, Figure 2A). When the animals were analyzed at the time points when all *Fth*^Δ*/*Δ^ mice were still alive, we could observe markedly elevated bacterial burdens in the spleen and liver as early as 12 hours after infection (Supplemental Figure 6C). Similarly, pathogen counts were highly significantly increased by more than one order of magnitude 20 hours after challenge (Figure 2B) in the iron-treated *Fth*^Δ*/*Δ^ as compared with the iron-administered WT cohort. Analogically, substantially higher bacterial colonization of inflammatory (iMac, CD11b^+^F4/80^+^) and resident macrophages (red-pulp macrophages and Kupffer cells, CD11b^–^F4/80^+^) of these organs could be ascertained in iron-treated KO mice (Figure 2, C and D).

Notably, *S*. Typhimurium primarily infects and is taken up by macrophages (29). Yet, the higher bacterial burden in *Fth*^Δ*/*Δ^ animals could not be explained by elevated phagocytic capabilities since in vivo uptake of latex beads by liver and spleen macrophages was not positively affected by the *Fth* genotype nor iron loading. Uptake of latex beads was significantly impaired in iron-loaded *Fth*^Δ*/*Δ^ mice in comparison with that in *Fth*^Δ*/*Δ^ mice (Supplemental Figure 4A). Importantly, we could exclude any differences in first line defense and uptake of *Salmonella*, as iron-loaded *Fth*^Δ*/*Δ^ mice infected i.v. with bacteria demonstrated comparable spleen and liver bacterial burden (Supplemental Figure 4B) and macrophage colonization (Supplemental Figure 4C) 3 hours after infection.

Taken together, deletion of FTH in myeloid leukocytes of iron-loaded *Fth*^Δ*/*Δ^ mice drastically reduced their survival. Notably, the unaltered bacterial burden and macrophage colonization 3 hours after infection let us speculate that primary defense mechanisms such as phagocytic exclusion of *Salmonella* or oxidative burst were not impaired in *Fth*^Δ*/*Δ^ animals and may not be the primary cause for this conspicuous phenotype.

### Myeloid FTH deficiency triggers uncontrolled cytokine response to Salmonella.

To investigate dysregulated signaling circuits during infection in iron-loaded myeloid-specific *Fth*-KO animals, we analyzed whole-genome gene expression in the spleens of *Fth^+/+^* and *Fth*^Δ*/*Δ^ animals challenged with/without i.v. iron and infected with 500 CFU *Salmonella* i.p. for 12 hours. Using 2-way ANOVA, we assessed the effect of iron and *Fth* genotype and, most importantly, the interaction of these 2 factors on expression of a particular gene (Supplemental Figure 5). Since the combination of the iron loading with the lacking myeloid *Fth* led to the drastically shortened survival, we assumed that the gene set regulated by the interaction of iron challenge and *Fth* genotype was most critical for this phenotype. By analyzing this iron/genotype interaction, we identified 271 genes significantly upregulated and 893 genes significantly downregulated in iron-loaded *Fth*^Δ*/*Δ^ mice as compared with iron-treated WT animals (Supplemental Table 1).

**Figure 3:**
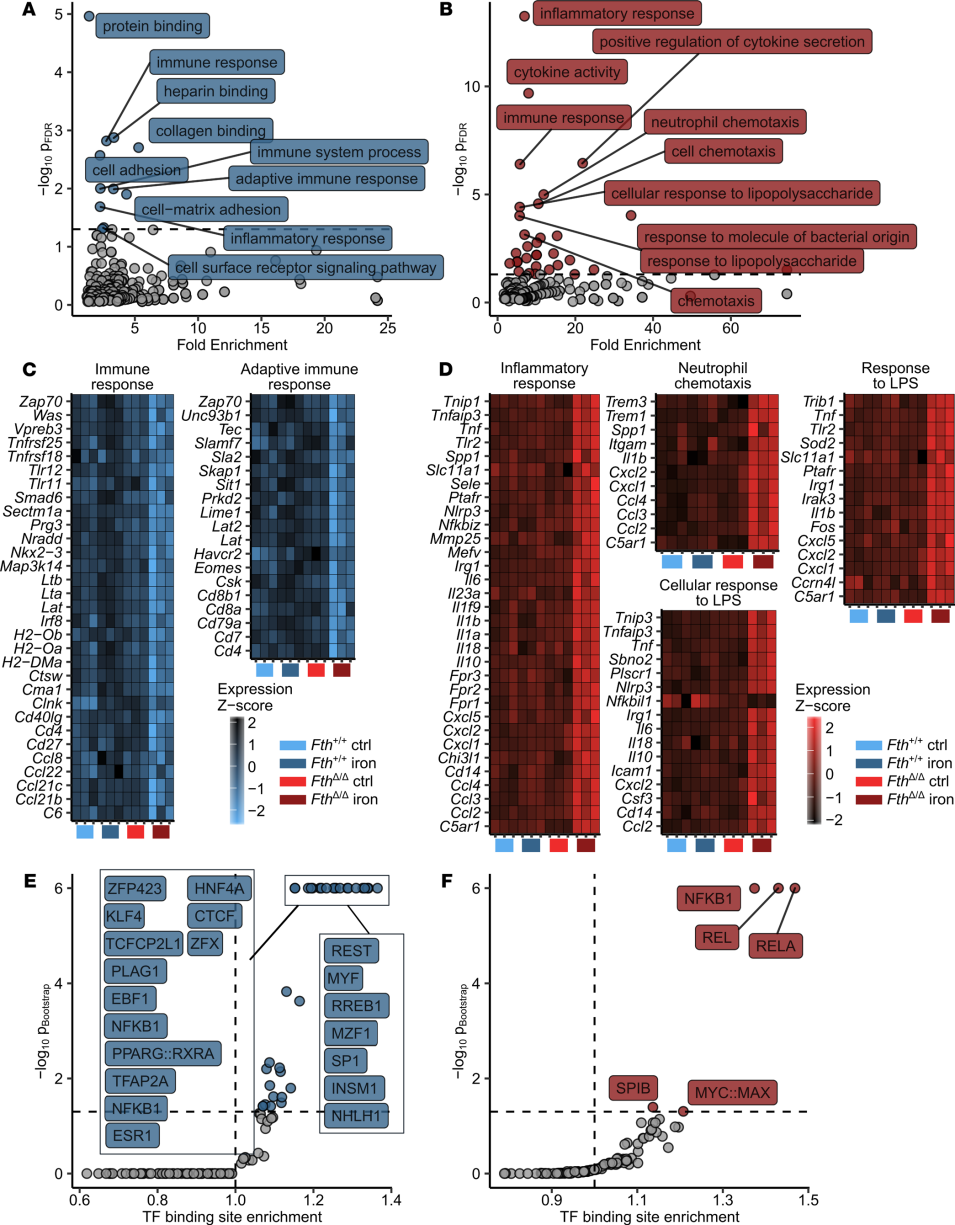
*S.* Typhimurium triggers unrestrained expression of proinflammatory NF-κB targets in iron-loaded *Fth^Δ/Δ^* mice. *Fth^fl/fl^* (*Fth^+/+^*) and *LysM-Cre Fth^fl/fl^* (*Fth*^Δ*/*Δ^) mice were i.v. administered PBS or iron isomaltoside (2 mg elementary Fe per animal) and infected 3 days later with 500 CFU GFP-expressing *S*. Typhimurium (STG) (*n* = 3 mice per group). Twelve hours after infection, total spleen RNA was isolated and subjected to a whole transcriptome measurement with gene microarrays. Genes significantly downregulated (*P*_ANOVA_ _iron/genotype_ < 0.05 and estimate_iron/genotype_ < -1.5, *n* = 893 genes) and upregulated (*P*_ANOVA_ _iron/genotype_ < 0.05 and estimate_iron/genotype_ > 1.5, *n* = 271 genes) were identified by 2-way ANOVA and linear regression as described in Methods and Supplemental Figure 5. For a list of significant genes with ANOVA *P* values and regression estimates, see Supplemental Table 1. (**A** and **B**) GO term enrichment analysis for genes significantly downregulated (**A**) and upregulated (**B**) by the iron/genotype interaction. Significant GO terms are highlighted in blue and red, respectively (downregulated genes: *n* = 10 significant GO terms, upregulated genes: *n* = 31 significant GO terms), 10 most significantly enriched GO terms are labeled with their names. For full results of GO term enrichment analysis, see Supplemental Tables 2 and 3. (**C** and **D**) Heatmap representation of normalized gene expression values (*z* score) for genes assigned to selected significantly enriched GO terms. (**C**) Significantly downregulated genes.(**D**) Significantly upregulated genes. Color scale corresponds to normalized expression. (**E** and **F**) Transcription factor (TF) binding site enrichment analysis for genes significantly downregulated (**E**) and upregulated (**F**) by the iron/genotype interaction. For each TF-binding motif, the Benjamini-Hochberg-corrected *P* value and fold enrichment are plotted. Significant TF-binding motifs are highlighted in blue and red (downregulated genes: *n* = 35, upregulated genes: *n* = 5 significant TF binding motifs), 10 most significantly enriched TF-binding motifs are labeled with their names.

Functional analysis of the gene set downregulated by the iron/genotype interaction using Gene Ontology (GO) term enrichment (Figure 3A and Supplemental Tables 2 and 3) revealed a substantial enrichment in genes involved in regulation of adaptive immune response such as *Cd4*, *Cd8a*, *Lat*, and *Zap70* (Figure 3C). In turn, a highly significant enrichment in genes linked to innate immune activation (*Tnfaip3*, *Itgam*, *C5ar1*, *Tlr2*, and *Tnf*), cytokine (*Il1b*, *Il6*, *Il10*, *Il18*, and *Tnf*), chemokine production (*Ccl2*, *Ccl3*, *Ccl4*, *Cxcl1*, *Cxcl2*, and *Cxcl5*), and TLR (*Tlr2* and *Cd14*), NF-κB (*Tnip,* *Tnfaip3*, and *Irak3*), and inflammasome signaling (*Nlrp3*, *Il1b*, and *Il18*) could be observed within the gene set upregulated in iron-treated FTH-deficient animals (Figure 3, B and D).

Next, we hypothesized that distinct transcription factors (TFs) may mediate the differences in gene expression as a function of the iron/genotype interaction. Therefore, we investigated possible common TF orchestrating the exacerbated transcriptional response to *Salmonella* in iron-loaded *Fth*^Δ*/*Δ^ animals (Figure 3, E and F, and Supplemental Tables 4 and 5). To this end, we compared the frequency of a particular TF binding site in the gene sets downregulated and upregulated by the iron/*Fth* interaction with the frequency in the whole genome. This analysis revealed a prominent overrepresentation of binding sites of various NF-κB isoforms (NF-κB, NFKB1, REL, and RELA) both in promoters of the downregulated and upregulated gene set, indicating that the aberrant iron turnover in iron-loaded *Fth*^Δ*/*Δ^ animals upon infection massively interferes with the NF-κB signaling pathway (Figure 3, E and F). Taken together, the combination of the increased iron loading with myeloid FTH deficiency triggers an excessive proinflammatory response to *Salmonella* manifested by, among others, an unharnessed NF-κB activation, cytokine, and chemokine production.

**Figure 4:**
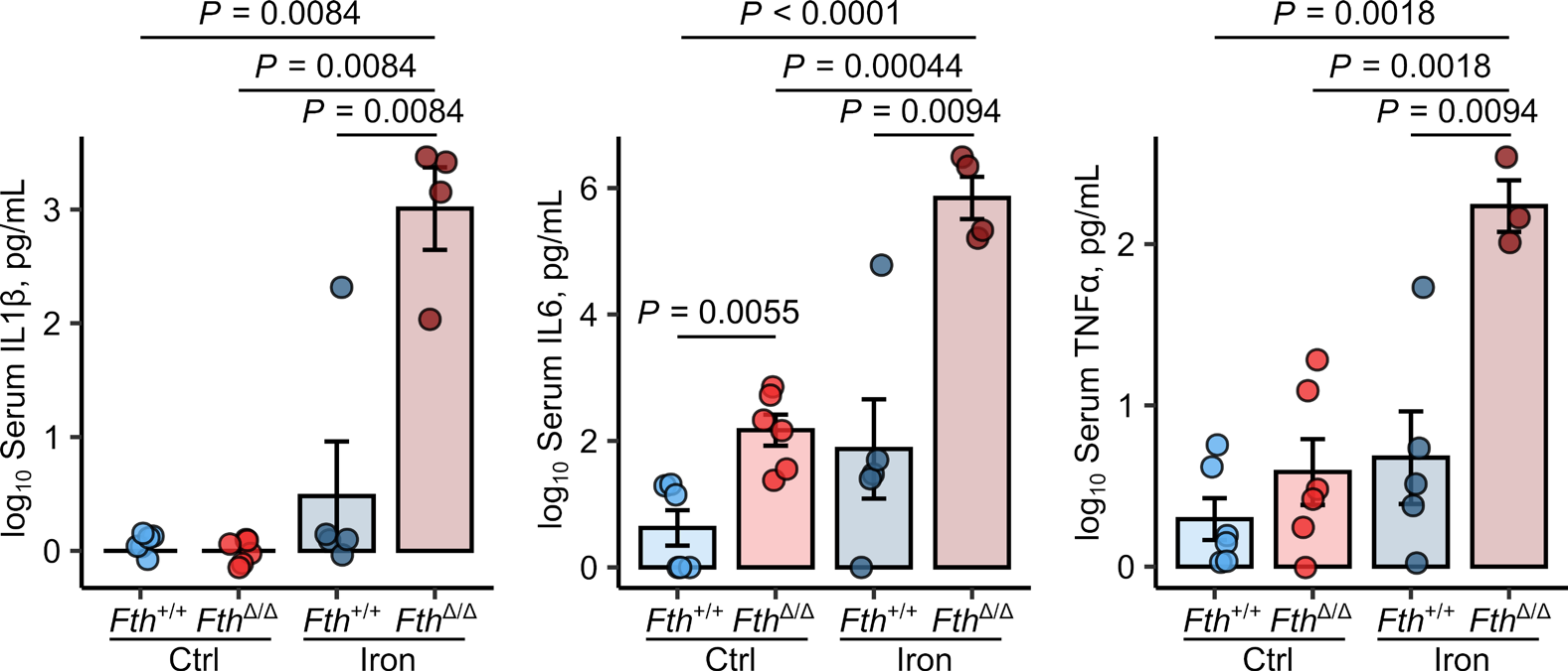
*S.* Typhimurium elicits unbraked cytokine and cellular innate response in iron-loaded *Fth^Δ/Δ^* mice. *Fth^fl/fl^* (*Fth^+/+^*) and *LysM-Cre Fth^fl/fl^* (*Fth*^Δ*/*Δ^) mice were i.v. administered PBS or iron isomaltoside (2 mg elementary Fe per animal) and infected 3 days later with 500 CFU GFP-expressing *S*. Typhimurium (STG). The animals were analyzed 20 hours after infection. Serum levels of IL-1β, IL-6, and TNF-α were measured by ELISA (*Fth^+/+^* ctrl: *n* = 6, *Fth^+/+^* iron: *n* = 5, *Fth*^Δ*/*Δ^ ctrl: *n* = 6, *Fth*^Δ*/*Δ^ iron: *n* = 4). Each point denotes single animal; bars with whiskers represent mean ± SEM. Statistical significance was assessed with 2-way ANOVA with Benjamini-Hochberg-corrected 2-tailed post hoc *t* tests. In the plots, post hoc test *P* values are indicated.

To further verify the previous results, we took a closer look at serum cytokine levels and infiltration of proinflammatory myeloid cells in the spleen and liver of iron-treated *Fth*^Δ*/*Δ^ mice infected with *Salmonella* for 12 and 20 hours. Twelve hours after infection, the levels of circulating inflammatory cytokines (IL-1β, IL-6, IL-18, and TNF-α) were still hardly detectable in most of the animals. Independent of iron status and genotype, we identified elevated *Il1b* and *Il6* transcript levels in the spleens of the iron-loaded *Fth*^Δ*/*Δ^ animals as compared with the remaining study groups (Supplemental Figure 6, A and B). In turn, a dramatic rise in IL-1β, IL-6, and TNF-α plasma concentrations was evident 20 hours after infection in i.v. iron-treated *Fth*-KO animals as compared with control and iron-administered WT animals and control-treated *Fth*^Δ*/*Δ^ mice (Figure 4).

Importantly, a quantitatively and qualitatively similar cytokine response to *Salmonella* in myeloid FTH-deficient mice could be elicited by iron loading with another clinically applicable i.v. iron preparation, iron gluconate, at the identical elementary iron dose (Supplemental Figure 7).

### Pharmacologic interference with the inflammasome/IL-1β pathway prevents the Salmonella-elicited cytokine storm in iron-loaded myeloid FTH-deficient mice.

Our microarray analysis pointed toward possible involvement of NF-κB and inflammasome signaling as well as myeloid cell recruitment in development of the uncontrolled inflammatory response in iron-treated and *Salmonella-*infected myeloid FTH-deficient mice (Figure 3, B, D, and F). To identify the functionally most relevant pathway, we first tested for NF-κB hyperactivity and treated control or iron-loaded *Fth^+/+^* and *Fth*^Δ*/*Δ^ animals with the canonical TLR/NF-κB signaling inducer LPS at a sublethal dose of 20 μg/kg (30). Animals were sacrificed 9 hours after the treatment, i.e., shortly after passing the peak of systemic inflammatory response defined by a body temperature drop (Supplemental Figure 7A). In this setting, iron-loaded *Fth*^Δ*/*Δ^ mice showed significantly decreased nadir body temperature (Supplemental Figure 8A) and substantially risen serum levels of IL-6 and TNF-α (Supplemental Figure 8B) compared with other experimental groups. However, the magnitude of the cytokine response to LPS did not reach the levels elicited by living *Salmonella* in the iron-challenged myeloid *Fth*^Δ*/*Δ^ (Supplemental Figure 7B and Figure 4) (*Salmonella*: 1.4 ± 0.7 × 10^6^ pg/mL IL-6 and 200 ± 80 pg/mL TNF-α; LPS: 4.2 ± 0.7 × 10^4^ pg/mL IL-6 and 70 ± 6 pg/mL TNF-α). Importantly, serum IL-1β, which was dramatically increased in iron-loaded *Fth*^Δ*/*Δ^ mice in *Salmonella* infection, was barely altered in the setting of LPS challenge.

However, even though some level of hypersensitivity of the NF-κB signaling pathway upon iron loading and myeloid FTH deficiency could be observed, it is unlikely that this mechanism solely underlies the dramatically increased cytokine response and mortality in iron-loaded *Fth*^Δ*/*Δ^ mice upon bacterial infection. This finding is further supported by the observation that the treatment of iron-loaded *Fth*^Δ*/*Δ^ mice, with a pharmacological NF-κB blocker BAY11-7082 (31) concomitantly with *Salmonella* infection, could neither reduce systemic cytokine production (Supplemental Figure 9A) nor improve infection control (Supplemental Figure 9B) and worsened the pathological phenotype.

**Figure 5:**
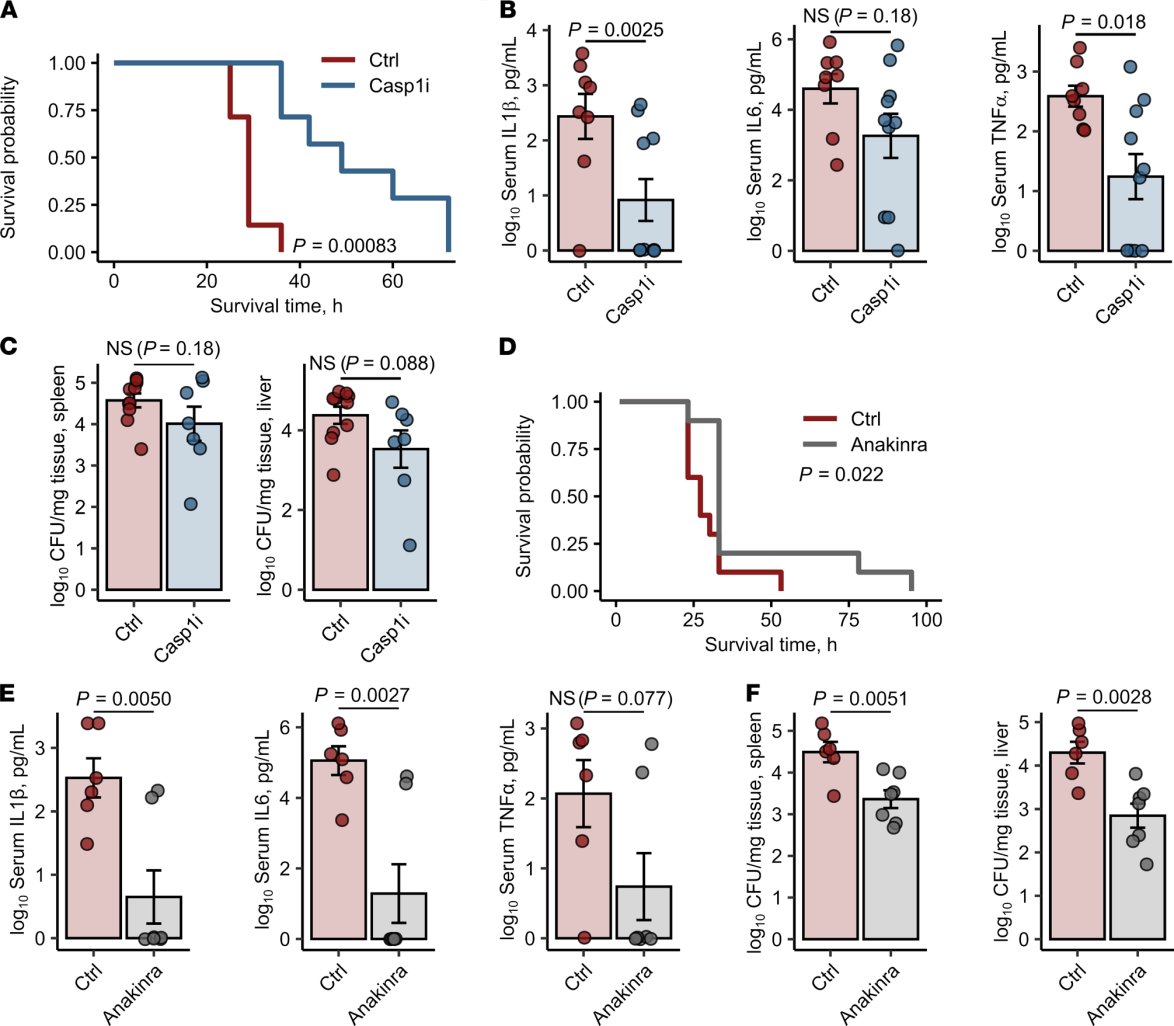
Inhibition of the inflammasome/IL-1β pathway prevents the *S.* Typhimurium–induced cytokine storm in iron-loaded *Fth^Δ/Δ^* mice. Fth^Δ/Δ^ mice were i.v. administered iron isomaltoside (2 mg elementary Fe per animal) and infected 3 days later with 500 CFU GFP-expressing *S*. Typhimurium (STG). Mice were i.p. injected with the caspase-1 inhibitor Ac-YVAD-cmk (Casp1i, 8 mg/kg, 1 hour after infection, **A–C**) or the IL-1β receptor antagonist anakinra (25 mg/kg, 3 hours after infection, **D–F**). The treatment with Ac-YVAD-cmk or anakinra was repeated in 12-hour intervals. Control mice were administered PBS. (**A**) Surviving animal fractions as a function of time (*n* = 7 mice per group). (**B**) Serum levels of IL-1β, IL-6, and TNF-α measured by ELISA 20 hours after infection (Ctrl: *n* = 10, Casp1i: *n* = 7). (**C**) Bacterial burden of the spleen and liver determined by plating of organ lysates (Ctrl: *n* = 10, Casp1i: *n* = 7). (**D**) Surviving animal fractions as a function of time (*n* = 10 mice per group). (**E**) Serum levels of IL-1β, IL-6, and TNF-α measured by ELISA 20 hours after infection (*n* = 7 per group). (**F**) Bacterial burden of the spleen and liver was determined by plating of organ lysates 20 hours after infection (*n* = 7 per group). In **A** and **D**, data are presented as Kaplan-Meier plots. In other panels, each point denotes single animal, and bars with whiskers represent mean ± SEM. In **A** and **D**, statistical significance was assessed with Wilcoxon test. In the other panels, statistical significance was assessed with 2-tailed *t* test. In the plots, Wilcoxon and *t* test *P* value are indicated.

To corroborate the prime effect of the inflammasome signaling on unharnessed cytokine response to *Salmonella*, we treated iron-loaded and *Salmonella-*infected *Fth*^Δ*/*Δ^ mice first with AC-YVAD-cmk, an oligopeptide inhibitor of the key common inflammasome component caspase-1. Administration of the inhibitor significantly prolonged survival of the animals and reduced the levels of circulating IL-1β and TNF-α and partly IL-6 at 20 hours after infection (Figure 5, A and B). However, this intervention only marginally improved infection control, as demonstrated by only slightly reduced bacterial loads of the spleen and liver (Figure 5C). To verify these promising results, we next administered the clinically applicable trap receptor for the key inflammasome-activated cytokine IL-1β, anakinra (25), to the infected iron-loaded myeloid FTH-deficient animals. By these means, we could significantly prolong survival (Figure 5D) and reduce not only the systemic levels of IL-1β but also of inflammasome-independent cytokines IL-6 and TNF-α as compared with iron-loaded *Fth*^Δ*/*Δ^ animals injected with PBS (Figure 5E). Importantly, systemic IL-1β neutralization significantly reduced bacterial burden in the liver and spleen as well (Figure 5F).

Taken together, the substantial reversal of the cytokine storm phenotype in iron-loaded *Fth*^Δ*/*Δ^ animals by interference with caspase-1 activity and the action of the inflammasome-dependent cytokine IL-1β activity strongly suggests that an exaggerated triggering of the inflammasome pathway represents the key effector mechanism driving lethal hyperresponsiveness to *Salmonella*.

### FTH deficiency in infected primary macrophages leads to overreactivity of the NF-κB and inflammasome signaling pathways and unharnessed cytokine secretion.

Given the complexity of the myeloid leukocyte compartment and its differential roles in hosting and eliminating *Salmonella* as well as cell-type-specific triggering of inflammatory pathways (32), we sought to verify the contribution of the NF-κB and inflammasome signaling to the cytokine hyperresponsiveness upon FTH deficiency of macrophages, the prime host cells for *Salmonella*.

To this end, we tested cytokine production capacities of primary peritoneal macrophages (PMs) derived from WT and *Fth*^Δ*/*Δ^ donors upon iron and *Salmonella* challenge in vitro. Notably, PMs were isolated from the mice without prior inflammatory stimulation in vivo, such as thioglycolate, to avoid any nonspecific priming of the cells. Surprisingly, FTH-deficient cells demonstrated high iron-independent production of IL-1β, IL-6, and TNF-α even without bacteria (Supplemental Figure 10A), indicating steady activity of proinflammatory signaling, most likely as a result of distorted iron metabolism (Figure 1). The secretion of the strictly inflammasome-dependent IL-1β (see the results of costimulation with AC-YVAD-cmk, Supplemental Figure 10B) was further strongly augmented by a short-time 3-hour *Salmonella* infection in those cells and was dramatically higher in the KO than in WT macrophages (Supplemental Figure 10A). Interestingly, production of IL-6 and TNF-α orchestrated in PMs primarily by NF-κB (see the results of costimulation with BAY11-7082; Supplemental Figure 10B) was stimulated by bacteria to a much lower extent in the *Fth*^Δ*/*Δ^ cells than in IL-1β. However, the levels of those cytokines upon *Salmonella* were significantly higher than in the WT cells (Supplemental Figure 10A). Although the fully fledged cytokine storm phenotype in *Fth*^Δ*/*Δ^ in vivo could only be observed in iron-loaded mice, production of the investigated cytokines was hardly boosted by iron; the significant but marginal effect of iron was found solely for IL-1β.

Collectively, at the level of the isolated macrophage, FTH deficiency goes hand in hand with uncontrolled spontaneous activation of the NF-κB and inflammasome signaling culminating in production of IL-1β, IL-6, and TNF-α. The latter signaling circuit is further augmented by *Salmonella*, leading to a dramatic increase of IL-1β secretion. At the systemic level, the labile iron-rich environment in the iron-loaded *Fth*^Δ*/*Δ^ mice further promotes pathogen growth, which in turn viciously amplifies the hyperreactive inflammatory NF-κB and inflammasome pathways causing fatal cytokine storm.

## Discussion

Ferritin is the most important intracellular iron storage protein. It is critically involved in maintaining iron homeostasis under steady-state situations and, even more importantly, in the case of infection. Intracellular free iron concentrations stimulate ferritin expression by stabilizing the mRNA via the iron response element/IRP system (33). As shown previously, infection with *Salmonella* or *Mycobacterium* spp. induces ferritin expression in macrophages (9, 34). However, less iron is incorporated into ferritin in the case of infection with an intracellular pathogen (9). Nevertheless, to date the molecular role of FTH in infection with intracellular pathogens has scarcely been investigated. Deletion of *Fth* and therefore, lack of ferroxidase activity, in the myeloid cell line should result in reduced iron storage capacities. We could show that the iron content of the spleen was decreased in myeloid FTH-deficient mice. This was in line with recently published results also showing impaired iron storage capacities in the spleen (24). Additionally, the expression of the iron export protein FPN1 and the labile iron pool was increased, which has been reported in FTH-deficient enterocytes (19).

Surprisingly, infection of myeloid FTH-deficient mice with *Salmonella* without additional iron loading resulted in unaltered survival or bacterial load as compared with WT animals. This is of interest, as a recent report with another intracellular bacterium, *Mycobacterium avium*, showed highly significantly increased susceptibility of myeloid FTH-deficient mice (24). Although both bacteria live intracellularly, there is a decisive difference concerning their exact localization: whereas *Mycobacterium* resides in the early phagosome, *Salmonella* primarily lives in the late phagosome (35). As we could show recently, the subcellular exact localization of intracellular pathogens matters (8). This might be one explanation for the pronounced phenotype in the *Mycobacterium* infection model.

However, by challenging the mice with an iron source used in clinics, we observed a significantly shortened survival and impaired infection control in myeloid FTH-deficient mice resulting in strongly elevated bacterial burden of the spleen and liver. In addition, we found a sustained upregulation of multiple inflammatory transcripts in iron-loaded *Fth*^Δ/Δ^ mice as early as 12 hours after infection and the resultant massive overproduction of the key proinflammatory cytokines IL-1β, IL-6, and TNF-α. Although, the cytokine storm poses the prime cause of mortality of the iron-administered myeloid FTH-deficient animals, as it could be inferred from the effects of AC-YVAD and anakinra treatments in vivo, we could not definitively state whether the increased bacterial loads further trigger the pathology or rather the impaired pathogen control results for a cytokine-mediated immunoparalysis. In light of published evidence and the analysis of the mice 12 hours after pathogen challenge, the first possibility seems to be the case. Specifically, multiple intracellular pathogens, including *Salmonella,* benefit from the elevated availability of the limiting microelement iron (23, 36) and such conditions are present in FTH-deficient macrophages. Therefore, these increased bacteria counts are likely to drive classical inflammatory pathways activating NF-κB (37) and inflammasome subtypes such as NLRC4 (38). However, we observed a strongly augmented inflammatory signaling in FTH-deficient primary macrophages even without any bacterial or proinflammatory stimulus and *Salmonella* infection could drastically boost secretion of the inflammasome-dependent IL-1β in these cells. Notably, both the labile iron accumulating in the *Fth*^Δ/Δ^ macrophages and ferritin on its own were postulated to affect NF-κB (39–42) and inflammasome signaling (43). Thus, under FTH deficiency and iron administration, the coincident accelerated pathogen growth providing a trigger for the inflammatory pathways along with the pronounced activation of the NF-κB and inflammasome signaling pose a self-perpetuating vicious cycle peaking at cytokine storm and death.

In line with the above hypothesis, administration of the caspase-1 inhibitor AC-YVAD-fmk (44) and the IL-1β trap receptor anakinra (45) effectively reduced cytokine levels in the iron-treated *Fth*^Δ/Δ^ animals and prolong their survival. In the case of anakinra, an improved pathogen control could be observed as well. This later effect was less accentuated in the AC-YVAD-fmk treatment, supposedly as a result of interference with caspase-1 — mediated pyroptosis being an important bacteria-clearing mechanism (38). Analogically, a negative interference with NF-κB microbial defense pathways may be the reason why the NF-κB inhibitor treatment was not able to reduce bacterial burden and to keep the pathogen-induced cytokine storm at bay in the iron-loaded myeloid FTH-deficient animals.

Taken together, we put forward a potentially novel link between FTH as a scavenger of intracellular labile iron, intracellular pathogen growth, and control of inflammatory response to infection. This might be especially relevant in patients with hereditary or acquired iron overload, such as poly-transfused patients, or patients with chemotherapy and severe infection or sepsis who have a higher risk for death in association with uncontrolled systemic iron homeostasis (46). Disrupted iron homeostasis has also been linked to adverse outcomes in patients with COVID-19 (47, 48), pointing to the general importance of iron homeostatic control for immune control and the course of infection (49). Of interest, anakinra has been applied for treatment of patients with severe COVID-19 who suffer from dysregulated hyperinflammatory immune response but also altered systemic iron trafficking (50–52). Future studies will have to clarify if such treatment will be of benefit for treatment of such infections with an exaggerated cytokine storm and whether part of this effect can be traced back to anakinra-mediated effects on iron homeostasis and associated induction of hyperinflammation as well as on iron delivery for microbes.

## Methods

### Bacteria and mice.

The SifB-GFP–expressing *Salmonella* *enterica* serovar *typhimurium* was described elsewhere (53) and used in all infection experiments.

*Fth*^Δ*/*Δ^ mice were generated by crossing the *LysM-Cre^+/+^* (JAX mice line B6.129P2-*Lyz2^tm1(cre)Ifo^*/J) and *Fth^fl/fl^* (JAX mice line B6.129-*Fth1^tm1.1Lck/^*J) animals and were gifted by Lukas Kühn (Ecole Polytechnique Fédérale de Lausanne, Lausanne, Switzerland). *Fth^+/+^* mice bred from the *Fth*^Δ*/*Δ^ line served as WT controls. Male mice (aged 8–12 weeks) were used in all experiments. Mice were kept in standard laboratory conditions and with standard rodent diet containing 180 mg elementary Fe/kg (SNIFF).

### Iron loading, Salmonella, and LPS treatment in vivo.

Iron loading was accomplished by an i.v. administration of iron isomaltoside (Medice) or i.p. injection of iron gluconate (Sanofi). The dose of each iron preparation was adjusted to 2 mg elementary Fe per animal. *Salmonella* infection or iron turnover analyses were conducted 72 hours after iron loading.

In most infection experiments, mice were i.p. administered 500 CFU *Salmonella*. Bacterial load in the spleen and liver was determined either by plating serial dilutions of organ homogenates on LB agar (Sigma-Aldrich) or by flow cytometry. For the cytometric load measurement, the absolute count of GFP-positive bacteria in the organ homogenate was determined with Precision Count Beads (BioLegend).

For the in vivo LPS challenge, mice were i.p. administered *Salmonella* LPS (Sigma-Aldrich) at a dose of 20 μg/kg.

In each in vivo assay, surface body temperature was measured in at least 12-hour intervals. Loss of reflexes (righting and grabbing reflex) and/or body temperature drop of the animal of more than 5°C compared with the preinfection baseline were deemed a humane endpoint for infection and survival experiments.

### In vivo blocking of NF-κB and IL-1β neutralization.

For NF-κB blocking, mice were i.p. injected with BAY11-7082 (10 mg/kg, Sigma-Aldrich) concomitantly with *Salmonella* infection.

For in vivo neutralization of IL-1, animals were i.p. treated with anakinra (25 mg/kg, Amgen) 3 hours after *Salmonella* infection. The anakinra treatment, which is known to antagonize murine IL-1β in experimental sepsis (54), was repeated in 12-hour intervals. Control animals were injected with PBS.

For in vivo selective and irreversible blocking of caspase-1, animals were infected with *Salmonella* as described. One hour later, we treated the animals with Ac-YVAD-cmk (8 mg/kg, Sigma-Aldrich) i.p. and repeated this treatment in 12-hour intervals. Control animals were injected with a 1% DMSO solution in water.

### Primary macrophage culture.

Macrophages were cultured in DMEM with 10% FCS (PAN Biotech), 2 mM L-glutamine (Lonza), and 1% penicillin-streptomycin (Lonza).

For BMDM differentiation, bone marrow cells were obtained from tibias and femurs flushed with cold PBS containing 1% penicillin-streptomycin. Bone marrow cells were cultured for 7 days in the presence of 50 ng/mL recombinant murine M-CSF (Preprotech).

For isolation of peritoneal exudate macrophages (PEM), peritoneal cavities of the mice without any inflammatory pretreatment were washed 3 times with 10 mL prewarmed PBS. PMs were incubated in DMEM with 10% FCS and antibiotics with/without 50 μM Fe^3+^ (iron III sulfate) for 3 hours and subsequently infected with logarithmic phase GFP-expressing *S*. Typhimurium at multiplicity of infection (MOI) of 10 CFU bacteria to 1 cell as described before (23). Gentamycin at 25 μg/mL (Thermo Fisher) was added 1 hour after infection to eliminate extracellular bacteria and supernatants collected at 3 hours after bacteria challenge.

### Flow cytometry, cellular labile iron pool measurement.

Spleen cells were isolated by meshing the organ through a 100 μm cell strainer (Corning) with PBS. Liver leukocytes were obtained from mechanically dissociated, Liberase TM/DNaseI digested organs (0.16 U/mL and 10 μg/mL, respectively, both from Sigma-Aldrich) essentially as described previously (55). Erythrocyte lysis was done with ACK buffer (150 mM NH_4_Cl, 10 mM KHCO_3_, and 0.1 mM Na_2_EDTA, all from Sigma-Aldrich). Flow cytometry staining was performed as described previously (56) with the following antibodies: anti-mouse CD45 (clone 3F11), CD11b (M1/70), Gr-1 (RB6-8C5), Ly6C (HK1.4), and F4/80 (BM8). Flow cytometry antibodies were purchased from BioLegend and Thermo Fisher. Organ-infiltrating leukocytes were identified as presented in Supplemental Figure 2. Total organ bacterial load was determined as described previously. Leukocyte population-specific infection with GFP-expressing *Salmonella* was measured as described previously (57) and presented in Supplemental Figure 2.

Measurement of cellular labile iron pool (LIP) was performed essentially as described previously (58, 59). Briefly, adherent macrophages were treated with 50 μM Fe^3+^ for indicated time points, harvested by scraping and stained with 1 μg/μL calcein AM (Thermo Fisher) for 5 minutes at 37°C. The difference between calcein fluorescence between the iron-untreated sample and the given iron-treated sample (ΔMFI calcein) is assumed to be proportional to cellular LIP size (59).

Flow cytometry measurement were conducted with the Gallios instrument (Beckman Coulter). Data analysis was accomplished with FlowJo software (BD).

### Determination of iron, hepcidin, nontransferrin-bound iron and cytokine concentration, lactate dehydrogenase activity, and Prussian Blue staining.

Liver and spleen tissues samples were dried, and their nonheme iron content was measured using the bathophenanthroline method as described previously (60). The results were calibrated to the protein content as determined with the BCA protein assay (Thermo Fisher). Plasma iron was determined with a kit from BioAssay Systems. Plasma hepcidin, IL-6, TNF-α, and IL-1β were determined with ELISA kits (hepcidin: Intrinsic Lifescience, all others: BD). Prussian Blue staining of formalin-fixed paraffin-embedded spleen and liver sections was performed as described previously (14).

### Quantitative real-time PCR and Western blotting.

RNA was isolated after lysis by PeqGold TriFast (Peqlab) followed by chloroform-phenol extraction. Reverse transcription was performed with M-MLV reverse transcriptase (Thermo Fisher) and random hexamer primers (Carl Roth) as described previously (8). Quantitative real-time PCR was performed with SoFast Eva Green master mix (BioRad) and primers, the sequences of which are listed in Supplemental Table 6. *Hprt* (hypoxanthine guanine phosphoribosyl transferase, Entrez Gene ID 15452) served as the house-keeping gene for expression normalization. Relative gene expression was calculated with the ΔCt method and presented as log_2_ expression, where log_2_ expression_gene_ = Ct_gene_ — Ct*_Hprt_*.

Protein extraction and Western blotting were performed exactly as described previously (61). We used the following antibodies: rabbit anti-mouse FTH1 (1:1000; Cell Signaling, 3998), rabbit anti-mouse Ferritin light chain (1:1000; Abcam, ab69090), mouse anti-human Transferrin receptor (1:1000; Invitrogen, PA1-84854), rabbit anti-human FPN1 (1:2000; Eurogentec, NRU 451443), and rabbit anti-human β-actin (1:500; Sigma-Aldrich, A2066).

### Iron uptake and release assay.

Iron uptake and release assays were essentially done as previously described (8). For the macrophage iron import assay, cells were cultured with 5 μM ^59^Fe-citrate (Perkin Elmer). At indicated time points, radioactivity of the culture supernatant was measured with a γ-counter (Perkin Elmer). For the iron release assay, cells were first incubated for 2 hours with 5 μM ^59^Fe-citrate, washed extensively, and cultured in non-radioactive medium. At indicated time points, radioactivity of the culture supernatant was determined with a gamma counter (Wallac, Perkin Elmer). For both assays, supernatant radioactivity was expressed as cpm normalized to whole-cell lysate protein concentration to account for differences in culture density.

### Whole-transcriptome analysis with microarrays.

*Fth^fl/fl^* (*Fth^+/+^*) and *LysM-Cre Fth^fl/fl^* (*Fth*^Δ*/*Δ^) mice were i.v. administered PBS or iron isomaltoside (2 mg elementary Fe per animal) and infected 3 days later with 500 CFU GFP-expressing *S*. Typhimurium (*n* = 3 per group). Twelve hours after infection, animals were sacrificed and whole-spleen RNA was isolated as described. RNA quality tested with RNA 6000 Nano Kit and Bioanalyzer 2100 (both from Agilent) and exceeded 8.2 RNA Integrity Number. Whole-genome expression was performed with Mouse Gene 2.0 ST Array (Thermo Fisher) following the manufacturer’s protocol.

Whole-transcriptome analysis was performed with R programming suite, version 3.6.1. Probe signals were normalized with the RMA algorithm (Robust Microarray Average), assigned to transcript identifiers and log_2_ expression levels calculated with package oligo (62). For identification of probes, the expression of which was significantly differentially regulated by the *Fth* genotype, iron loading, and the iron/genotype interaction 2-way ANOVA and factorial linear regression was applied. Genes significantly downregulated (*P*_ANOVA_ _iron/genotype_ < 0.05 and regression estimate_iron/genotype_ < –log_2_ 1.5, *n* = 893 genes) and upregulated (*P*_ANOVA_ _iron/genotype_ < 0.05 and regression estimate_iron/genotype_ > 1.5, *n* = 271 genes) by were further investigated. For a list of significant genes with ANOVA *P* values and regression estimates see Supplemental Table 1. Mouse microarray data can be accessed from the Gene Expression Omnibus (GSE145114).

GO enrichment analysis for the significantly regulated genes was performed with DAVID version 6.8 (63). For full results of GO term enrichment analysis for the downregulated and upregulated genes, see Supplemental Tables 2 and 3.

TF binding site enrichment analysis for the significantly regulated genes was performed with an in-house written R script (https://github.com/PiotrTymoszuk/TF-enrichment). First, TF binding (for all TF represented in the JASPAR and TRANSFAC databases) in the promoters of genes of interest and in the whole mouse transcriptome was predicted with D-Light with the default program settings (64). Next, for a particular TF, the total count of binding sites in the gene set of interest (containing *n* genes) was compared with the total count of binding sites in 10^6^ randomly generated gene sets (containing n random genes) from the whole mouse genome. To obtain the *P* value for enrichment significance (*P*_bootstrap_), the number of random gene sets with the binding site count exceeding the binding site count in the gene set of interest was determined and divided by the number of random gene sets. *P*_bootstrap_ was adjusted for multiple comparisons with the Benjamini-Hochberg method. Adjusted *P* values of less than 0.05 were considered significant. For full results of TF-binding site enrichment analysis for the downregulated and upregulated genes, see Supplemental Tables 4 and 5.

### Statistics.

Statistical data analysis and result visualization was performed with R programming suite (version 4.0.3, tidyverse package bundle). Details on microarray data analysis are mentioned above. All other data were analyzed with 2-tailed *t* tests, 1- and 2-way ANOVA, and/or factorial linear regression, as indicated in the figure legends. Post hoc testing was accomplished with Benjamini-Hochberg adjusted 2-tailed *t* tests. Strongly nonnormal distributed variables (Shapiro-Wilk test and visual inspection of qq plots) were log_10_ transformed prior to analysis. Mouse survival data were analyzed with Cox regression and Kaplan-Meier method using Wilcoxon’s test. If not indicated otherwise, data are presented as dot plots, where each point represents a single observation. Data are shown as the mean ± SEM. A *P* value of less than 0.05 was considered significant.

### Study approval.

All animal experiments were performed in accordance with the Austrian Experimental Animal Welfare Act 2012 (Tierversuchsgesetz 2012) and were approved by the Federal Ministry of Science and Education (approval no. BMWF-66.011/0142-WF/V/3b/2014, Vienna, Austria).

## Author contributions

DH and PT conceived the project, designed and performed experiments, analyzed and interpreted data, and wrote the manuscript. VP, RH, SD, SS, ED, SB, MS, and AMM performed experiments. DB and MN provided intellectual input. IT and GW conceived the project, designed experiments, analyzed and interpreted data, and wrote the manuscript.

## Supplementary Material

Supplemental data

Supplemental Table 1

Supplemental Table 2

Supplemental Table 3

Supplemental Table 4

Supplemental Table 5

## Version 1 07/08/2021

Electronic publication
