# Supplementary material for: Ferritin H deficiency deteriorates cellular iron handling and worsens Salmonella typhimurium infection by triggering hyperinflammation
Source: JCI Insight. 2021 Jul 8;6(13):e141760. doi: 10.1172/jci.insight.141760 (PMC8410025; doi:10.1172/jci.insight.141760)
Supplement: Supplemental data [file jciinsight-6-141760-s199.pdf]

Supplementary Material

Ferritin H deficiency deteriorates cellular iron handling and worsens *Salmonella* Typhimurium infection by triggering hyperinflammation

David Haschka[1*], Piotr Tymoszuk[1, 5*], Verena Petzer[1], Richard Hilbe[1], Simon Heeke[1], Stefanie Dichtl[1], Sergej Skvortsov[2], Egon Demetz[1], Sylvia Berger[1], Markus Seifert[1], Anna-Maria Mitterstiller[1], Patrizia Moser[3], Dirk Bumann[4], Manfred Nairz[1], Igor Theurl[1*], Guenter Weiss[1*]

[1] Department of Internal Medicine II, Medical University of Innsbruck, Anichstraße 35, A – 6020 Innsbruck, Austria.

[2] Department of Therapeutic Radiology and Oncology, EXTRO-Lab, Tyrolean Cancer Research Institute, Medical University of Innsbruck, Anichstraße 35, A – 6020 Innsbruck, Austria.

[3] Institute of Pathology, INNPATH GmbH, Anichstraße 35, A – 6020 Innsbruck, Austria.

[4] Biozentrum, University of Basel, Klingelbergstrasse 50/70, CH – 4056, Basel, Switzerland.

[5] current affiliation: daas.tirol, Tiergartenstraße 20 D12, A - 6020 Innsbruck

* authors contributed equally

Supplementary Table S1

Supplementary Table S1. Table of transcripts significantly regulated by the iron: genotype interaction. Transcripts significantly regulated by iron: genotype interaction in course of S. tm infection were identified as described in Figure 3, Materials and Methods and Supplementary Figure S5.. RefSeqID: NCBI RefSeq transcript identifier, HGNC Symbol: gene symbol, $F_{interaction}$: F statistics value for the iron: genotype interaction, $P_{ANOVA\ interaction}$: P value for the iron: genotype interaction, log_2 $Estimate_{interaction}$: linear regression estimate for the iron: genotype interaction. The table is available online.

Supplementary Table S2

Supplementary Table S2. Results of GO term enrichment analysis for transcripts downregulated by the iron: genotype interaction. Transcripts significantly regulated by iron: genotype interaction in course of S. tm infection were identified as described in Figure 3, Materials and Methods and Supplementary Figure S5. Gene Ontology (GO) term enrichment analysis for transcripts found downregulated by the iron: genotype interaction was performed with DAVID (Database for Annotation, Visualization and Integrated Discovery). GO Term ID: Gene Ontology term identifier, GO Term Name: Gene Ontology term name, Fold Enrichment: fold enrichment over GO term occurrence in mouse transcriptome, Raw P: raw Fisher test p value for significant GO term enrichment, P FDR: Benjamini-Hochberg-corrected Fisher test p value for significant GO term enrichment. The table is available online.

Supplementary Table S3

Supplementary Table S3. Results of GO term enrichment analysis for transcripts upregulated by the iron: genotype interaction. Transcripts significantly regulated by iron: genotype interaction in course of S. tm infection were identified as described in Figure 3, Materials and Methods and Supplementary Figure S5. Gene Ontology (GO) term enrichment analysis for transcripts found upregulated by the iron: genotype interaction was performed with DAVID (Database for Annotation, Visualization and Integrated Discovery). GO Term ID: Gene Ontology term identifier, GO Term Name: Gene Ontology term name, Fold Enrichment: fold enrichment over GO term occurrence in mouse transcriptome, Raw P: raw Fisher test p value for significant GO term enrichment, P FDR: Benjamini-Hochberg-corrected Fisher test p value for significant GO term enrichment. The table is available online.

Supplementary Table S4

Supplementary Table S4. Results of transcription factor binding site enrichment in the set of genes downregulated by the iron: genotype interaction. Transcripts significantly regulated by iron: genotype interaction in course of S. tm infection were identified as described in Figure 3, Materials and Methods and Supplementary Figure S5. Transcription factor (TF) binding site enrichment in the set of genes found downregulated by the iron: genotype interaction over the mouse whole-genome occurrence was investigated by a bootstraping procedure described in Materials and Methods. TF ID: Transfac database binding site identifier, TF symbol: Transfac database TF symbol, Enrichment: fold enrichment over binding site occurrence in mouse genome, Raw P: raw bootstrap test p value, P FDR: Benjamini-Hochberg-corrected raw bootstrap test p value. The table is available online.

Supplementary Table S5

Supplementary Table S5. Results of transcription factor binding site enrichment in the set of genes upregulated by the iron: genotype interaction. Transcripts significantly regulated by iron: genotype interaction in course of S. tm infection were identified as described in Figure 3, Materials and Methods and Supplementary Figure S5. Transcription factor (TF) binding site enrichment in the set of genes found upregulated by the iron: genotype interaction over the mouse whole-genome occurrence was investigated by a bootstraping procedure described in Materials and Methods. TF ID: Transfac database binding site identifier, TF symbol: Transfac database TF symbol, Enrichment: fold enrichment over binding site occurrence in mouse genome, Raw P: raw bootstrap test p value, P FDR: Benjamini-Hochberg-corrected raw bootstrap test p value.

Supplementary Table S6

Supplementary Table S6. Primer sequences used in the study. Ensembl ID: Ensembl identifiers of detected transcripts, HGNC Symbol: HGNC gene symbol.

Ensembl ID	HGNC Symbol	Forward primer	Reverse primer
ENSMUST00000108518, ENSMUST00000048514	*Nlpr1a*	GGAAGGTACAGCAGGGACAT	CGCTTTCTCAGGGTCGTACA
ENSMUST00000108516, ENSMUST00000108515, ENSMUST00000108514, ENSMUST00000094046, ENSMUST00000136493	*Nlrp1b*	ATGACCTGTGTGACGATGGT	TCCATGTTCTCAGTGGGGAC
ENSMUST00000023238	*Gsdmd4*	GGTTCTGGAAACCCCGTTAT	ATTCATGGAGGCACTGGAAC
ENSMUST00000027015	*Casp1*	CCAGGCAAGCCAAATCTTTA	AGTCCTGGAAATGTGCCATC
ENSMUST00000162846, ENSMUST00000027012	*Casp11*	ACAATGCTGAACGCAGTGAC	CTGGTTCCTCCATTTCCAGA
ENSMUST00000033056, ENSMUST00000205594	*Pycard*	ACAGAAGTGGACGGAGTGCT	CTCCAGGTCCATCACCAAGT
ENSMUST00000147604, ENSMUST00000166137	*Aim2*	AGATTGCCAGGAGCACACTC	GAGGCAGCAGAGCAGTTTTC
ENSMUST00000026845 ENSMUST00000195978 ENSMUST00000199183 ENSMUST00000199765	*Il6*	TGTTCTCTGGGAAATCGTGGA	AAGTGCATCATCGTTGTTCAT ACA
ENSMUST00000028881 ENSMUST00000141979 ENSMUST00000155994	*Il1b*	GATGAGGACATGAGCACCTTC TT	GCAGGTTATCATCATCATCCC A
ENSMUST00000025263 ENSMUST00000167924	*Tnfa*	TTCTATGGCCCAGACCCTCA	TTGCTACGACGTGGGCTACA
ENSMUST00000076470	*Nlrp4e*	GCCTGGGATATGACCTTCAA	CTTCTTTCCAGTCGCCTTTG
ENSMUST00000101148, ENSMUST00000079476, ENSMUST00000149126	*Nlrp3*	CATGGCTGTGTGGATCTTTG	CAGCAAACCCATCCACTCTT
ENSMUST00000052124	*Nlrc4*	GGCCTGCAACCTCTTTCTTA	CGATGGTCCTTCTTCCACAT

Supplementary Figure S1

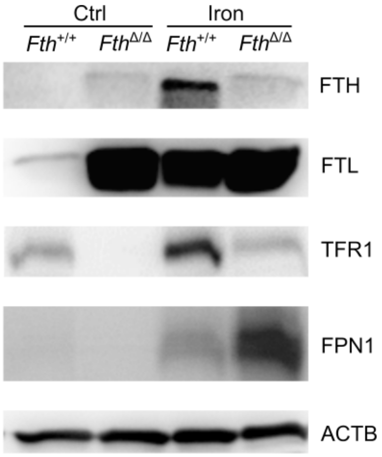

Efficient FTH deletion in *Fth*$^{\Delta/\Delta}$ bone marrow-derived macrophages.
Bone marrow-derived macrophages were differentiated from *Fth*$^{flox/flox}$ (*Fth*$^{+/+}$) and *LysM-Cre Fth*$^{fl/fl}$ (*Fth*$^{\Delta/\Delta}$) bone marrow and stimulated with vehicle or 50 µM Fe^{3+} ($FeCl_3$) for 12 hours. Protein levels of ferritin heavy chain (FTH), ferritin light chain (FTL), transferrin receptor 1 (TFR1) and ferroportin (FPN1) were assessed by Western Blotting. β-actin served as loading control. Results of a representative experiment out of two (n = 2) performed are shown.

Supplementary Figure S2

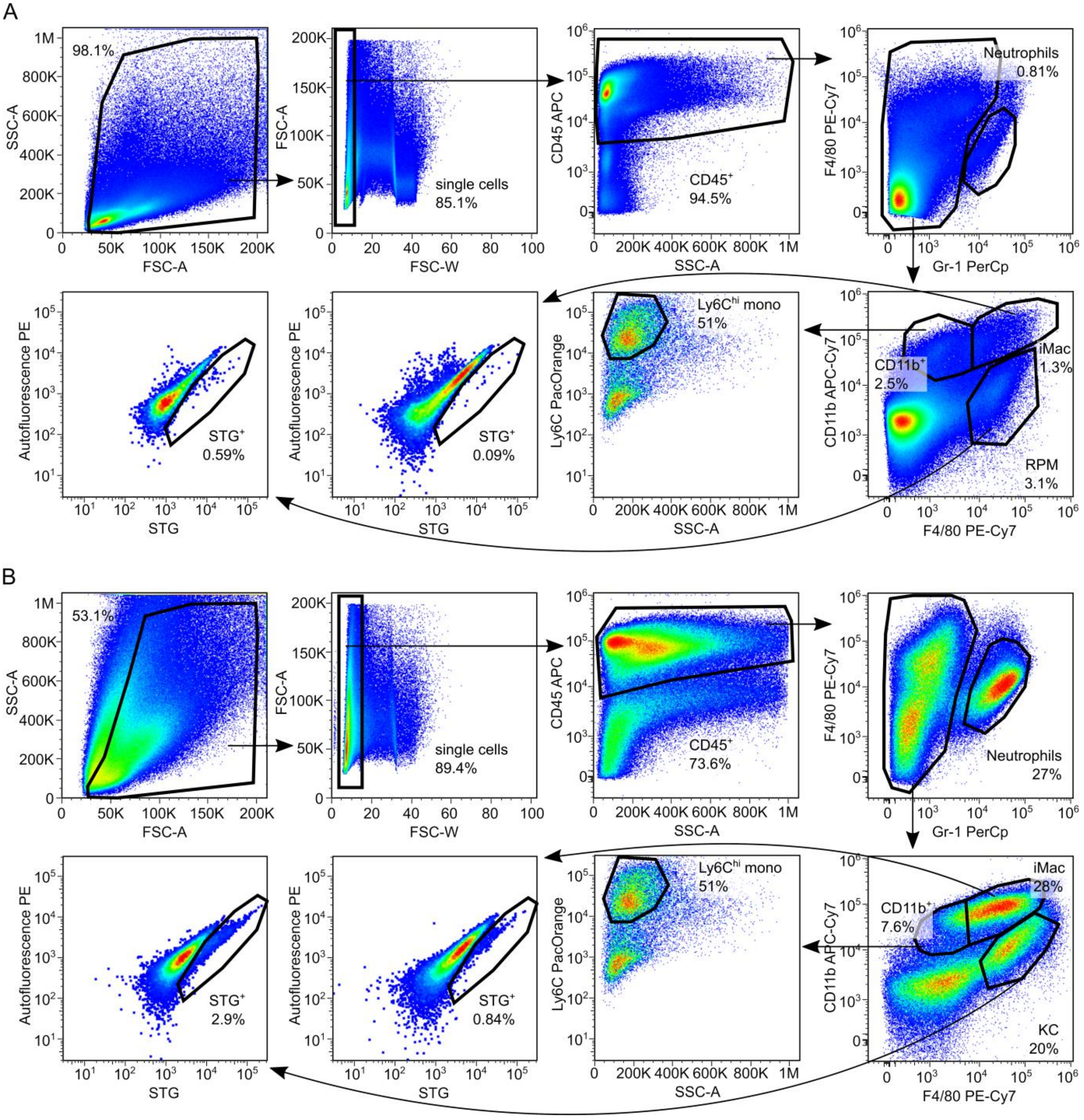

Flow cytometry gating strategy used for identification of splenic and hepatic myeloid cells. Determination of leukocyte population-specific bacterial load.

(A, B) Pan-leukocytes were defined as $CD45^+$ cells. Neutrophils were defined as $CD45^+$ $F4/80^-$ $Gr\text{-}1^+$, $Ly6C^{hi}$ monocytes as $CD45^+$ $Gr\text{-}1^-$ $F4/80^-$ $CD11b^+$ SSC^{lo} $Ly6C^{hi}$, inflammatory macrophages (iMac) as $CD45^+$ $Gr\text{-}1^-$ $CD11b^+$ $F4/80^+$, resident macrophages (red pulp macrophages of the spleen: RPM and Kupffer cells of the liver: KC) as $CD45^-$ $Gr\text{-}1^-$ $CD11b^-$ $F4/80^+$ leukocytes. Percent of GFP *Salmonella*-positive leukocytes (STG^+) was determined by as presented with dot plots showing GFP fluoresecence

plotted against PE-like autofluorescence. Representative results of flow cytometry analysis of a S.tm-infected, iron-loaded *LysM-Cre* $Fth^{fl/fl}$ ($Fth^{\Delta/\Delta}$) mouse are presented: (A) spleen, (B) liver.

Supplementary Figure S3

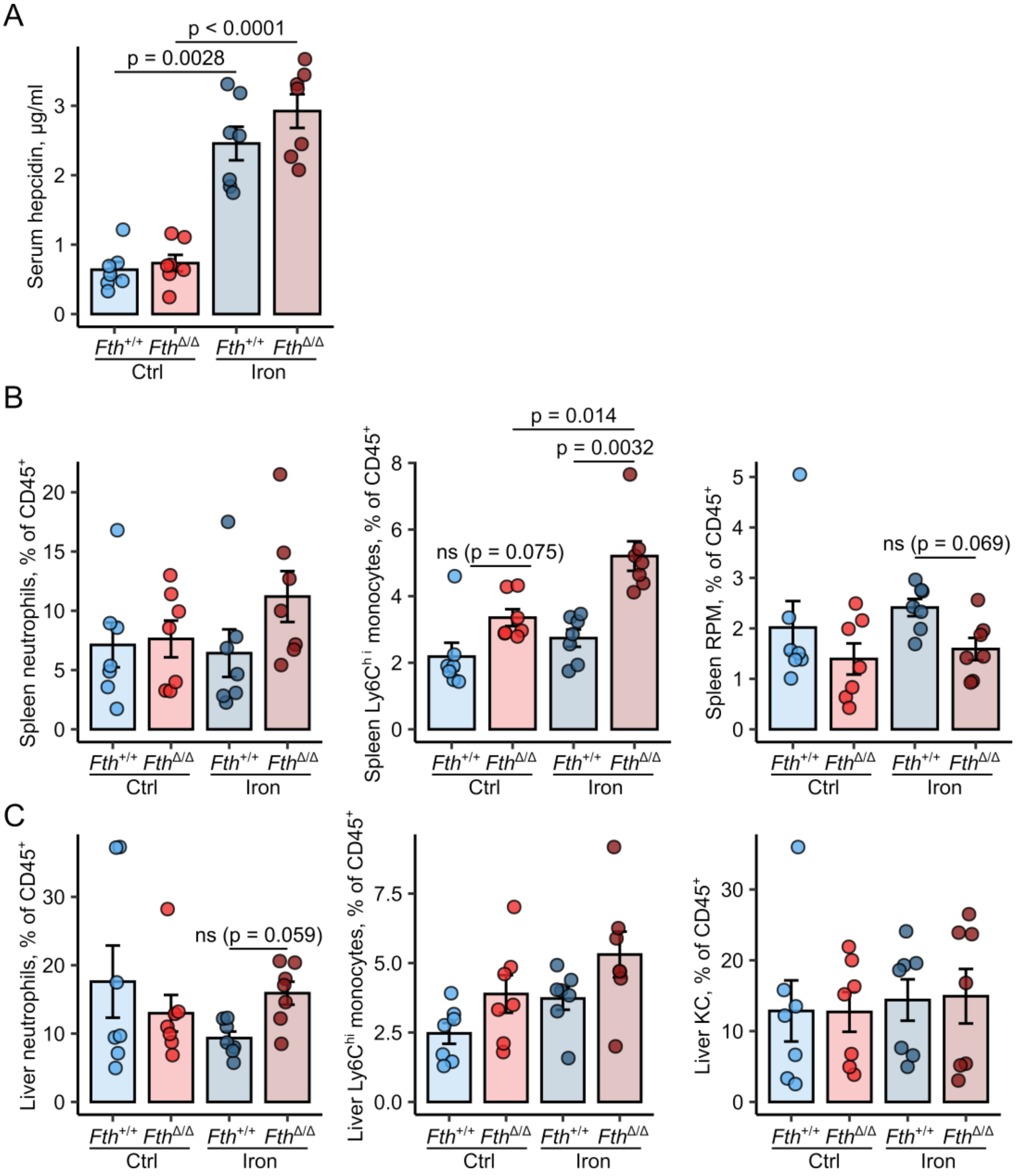

Macrophage FTH deficiency does not affect systemic hepcidin induction and organ leukocyte composition.

Fth$^{flox/flox}$ (*Fth*$^{+/+}$) and *LysM-Cre Fth*$^{fl/fl}$ (*Fth*$^{\Delta/\Delta}$) mice mice were injected intravenously with PBS or iron isomaltoside (2 mg elementary Fe) and analyzed three days later (n = 7 mice per group).

(A) Serum hepcidin levels measured by ELISA.

(B, C) Composition of splenic (B) and hepatic (C) myeloid cell compartment. Cellularities of neutrophils, Ly6C^{hi} monocytes, resident red pulp macrophages (RPM) and Kupffer cells (KC) were determined by flow cytometry and expressed as percent of CD45^{+} cells.

Each point denotes single animal, bars with whiskers represent means ± SEM. Statistical significance was assessed with two-way ANOVA with Benjamini-Hochberg-corrected two-tailed post-hoc T tests. In the plots, post-hoc test p values are indicated.

Supplementary Figure S4

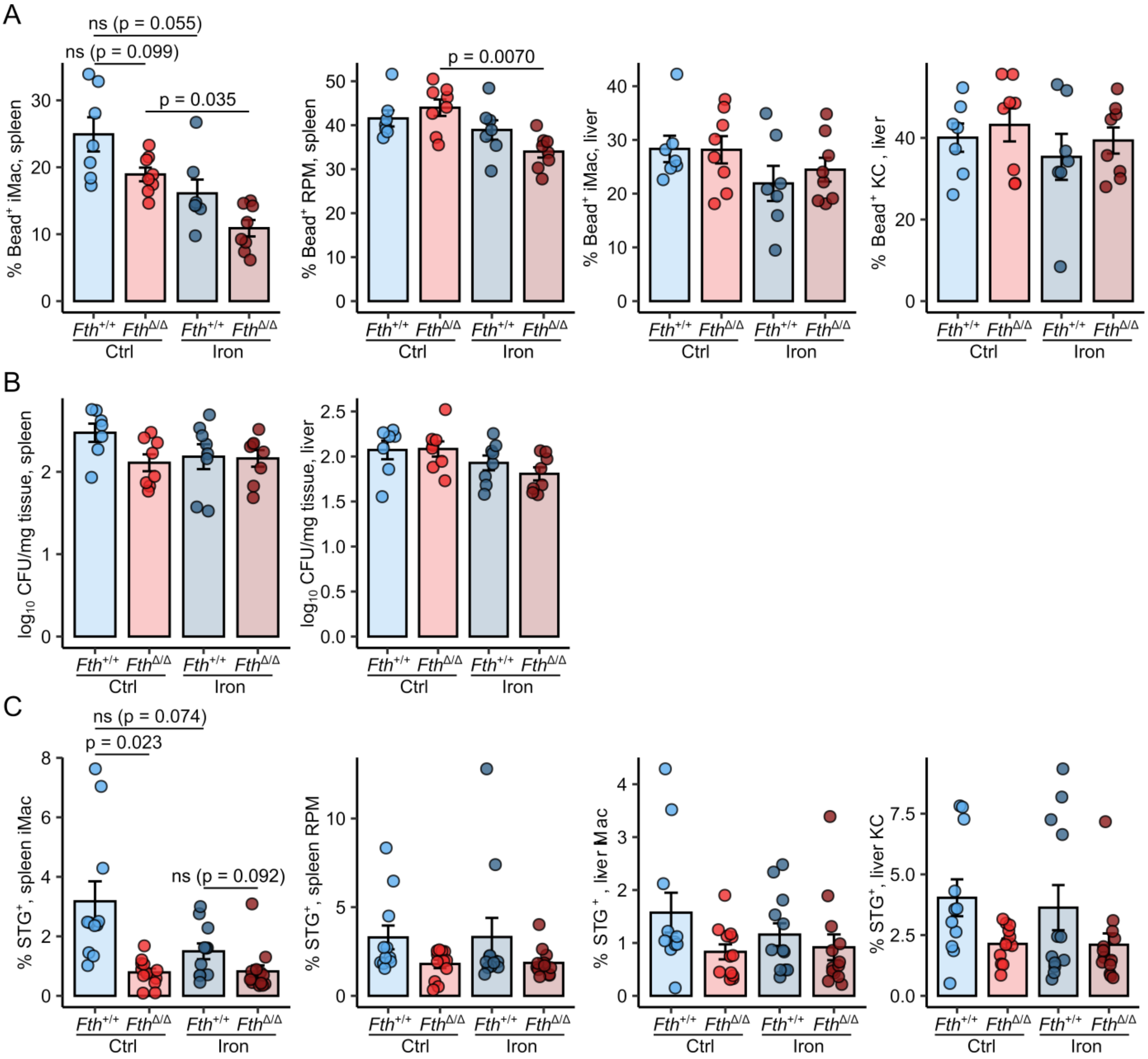

Unimpaired phagocytic capacities and bacterial defense of FTH- deficient macrophages in vivo. (A) *Fth*$^{flox/flox}$ (*Fth*$^{+/+}$) and *LysM-Cre Fth*$^{fl/fl}$ (*Fth*$^{\Delta/\Delta}$) mice were intravenously loaded with iron isomaltoside (2 mg elementary Fe per animal) and intravenously injected with YG Fluorescebrite beads (1:25 dilition, 200 µl per animal) three days later (*Fth*$^{+/+}$ ctrl: n = 7, *Fth*$^{+/+}$ iron: n = 7, *Fth*$^{\Delta/\Delta}$ ctrl: n = 8, *Fth*$^{\Delta/\Delta}$ iron: n = 8). Bead phagocytosis by splenic and hepatic inflammatory macrophages (iMac) and resident macrophages (spleen: red pulp macrophages, RPM, liver: Kupffer cells, KC) was analyzed by flow cytometry 3 hours post bead administration and expressed as percent of bead-positive cells within the parent population.

(B, C) *Fth*$^{+/+}$ and *Fth*$^{\Delta/\Delta}$ mice were intravenously loaded with iron isomaltoside (2 mg elementary Fe per animal) and intravenously infected with 10^8 CFU GFP-expressing S.tm (STG) per animal three days later. Animals were analyzed 3 hours post infection. (B) Bacterial burden of the spleen and liver determined by flow cytometry of organ lysates (*Fth*$^{+/+}$ ctrl: n = 7, *Fth*$^{+/+}$ iron: n = 8, *Fth*$^{\Delta/\Delta}$ ctrl: n = 8, *Fth*$^{\Delta/\Delta}$ iron: n = 8). (C) Bacterial colonization of splenic and hepatic inflammatory (iMac) and resident macrophages (spleen: red pulp macrophages, RPM, liver: Kupffer cells, KC) measured by flow cytometry and

expressed as percent of STG-positive cells within the parent population (*Fth*$^{+/+}$ ctrl: n = 11, *Fth*$^{+/+}$ iron: n = 11, *Fth*$^{Δ/Δ}$ ctrl: n = 12, *Fth*$^{Δ/Δ}$ iron: n = 13).

Each point denotes single animal, bars with whiskers represent means ± SEM. Statistical significance was assessed with two-way ANOVA with Benjamini-Hochberg-corrected two-tailed post-hoc T tests. In the plots, post-hoc test p values are indicated.

Supplementary Figure S5

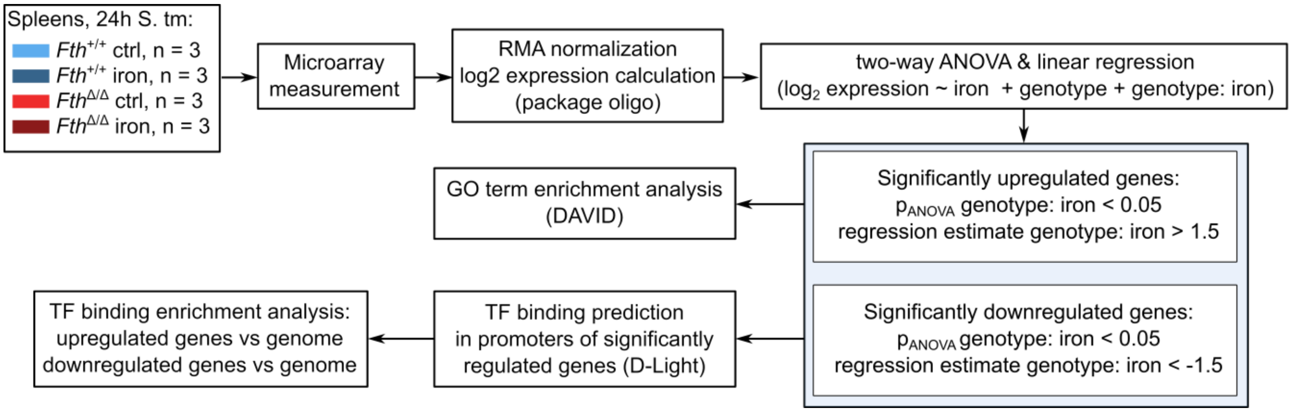

Strategy of whole-transcriptome data analysis.

*Fth^flox/flox^* (*Fth^+/+^*) and *LysM-Cre Fth^fl/fl^* (*Fth^Δ/Δ^*) mice were intravenously administered PBS or iron isomaltoside (2 mg Fe) and infected three days later with 500 CFU GFP-expressing S.tm (STG). 12 hours post infection, total spleen RNA was isolated and subjected to a whole transcriptome measurement with gene microarrays (Mouse Gene 2.0 ST Array). Probe signal intensities were RMA (Robust Multiarray Average) normalized, probes allocated to gene identifiers and log_2 expression calculated with the Bioconductor package oligo. For each gene, two-way ANOVA with the genotype, iron and genotype: iron interaction terms was performed, p values were calculated and adjusted with the Benjamini-Hochberg method. Genes with adjusted $p < 0.05$ for the genotype: iron interaction term were deemed significant. The gene set significantly regulated by the genotype: iron interaction was subjected to hierarchical clustering (average linkage algorithm, Euclidean distance, Genesis software), visualized as a heat-map and the cluster of genes significantly upregulated and downregulated in iron-loaded *Fth*$^{\Delta/\Delta}$ mice were identified.

To identify functional relationships for the genes significantly upregulated in iron-loaded *Fth*$^{\Delta/\Delta.}$ mice, gene ontology (GO) term enrichment analysis was performed with the DAVID 6.8 online tool.

To identify common transcription regulatory pathways for the genes significantly upregulated in iron-loaded *Fth*$^{\Delta/\Delta.}$ mice, transcription factor (TF) binding site enrichment analysis was performed with an in-house written R script. In brief, the total number of predicted binding sites of the particular TF (D-Light) in promoters of the significantly regulated gene set was compared with numbers of TF binding sites in 10^6 random gene sets from the entire mouse genome. For details see Materials and Methods.

Supplementary Figure S6

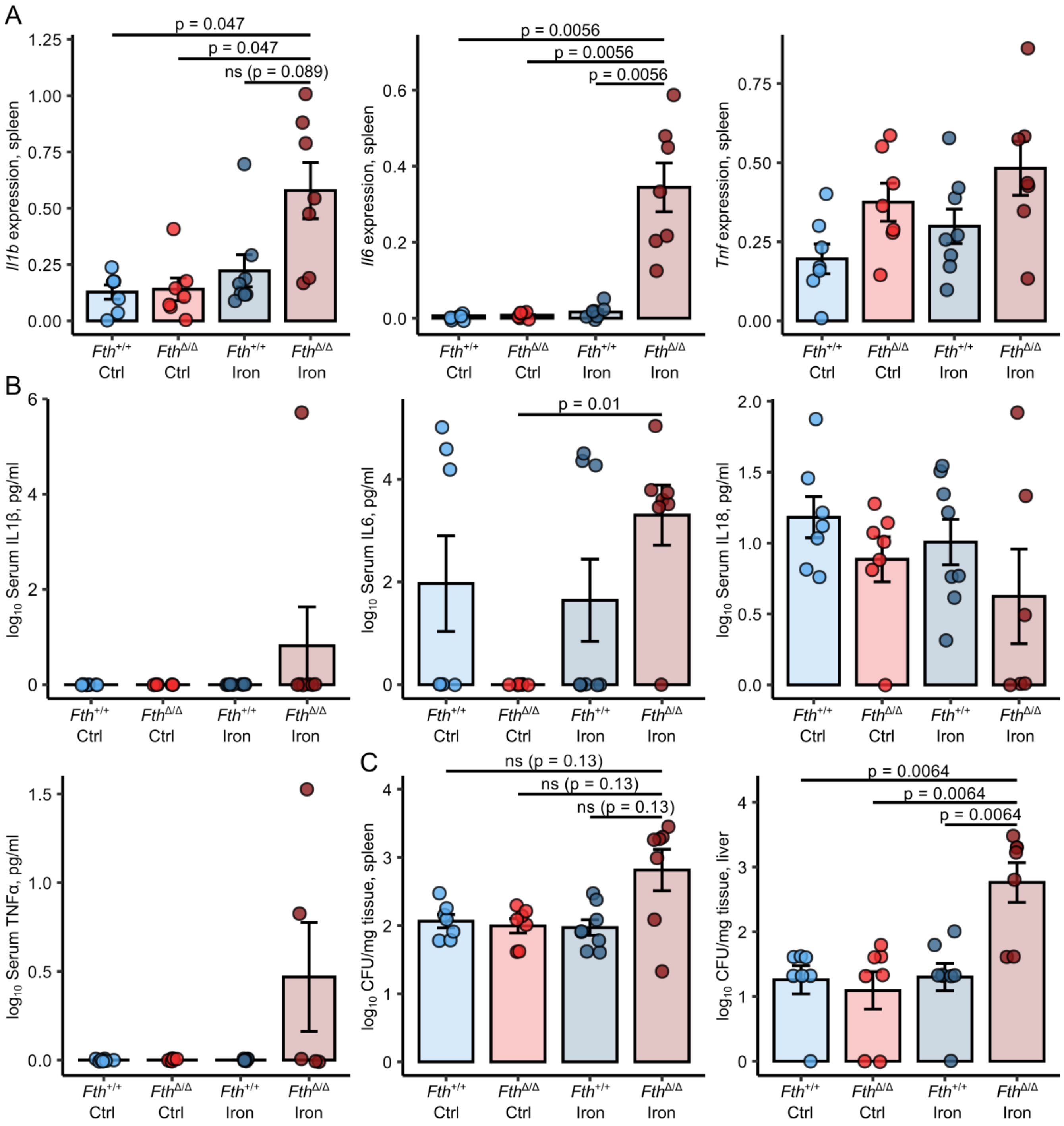

Spleen mRNA levels and serum concentrations of IL1β, IL6, IL18 and TNFα and bacterial loads in wildtype and myeloid FTH-deficient mice infected with *Salmonella* for 12 hours.

Fth$^{flox/flox}$ (*Fth*$^{+/+}$) and *LysM-Cre Fth*$^{fl/fl}$ (*Fth*$^{\Delta/\Delta}$) mice (n = 7) were intravenously administered PBS or iron isomaltoside (2 mg Fe) and infected three days later with 500 CFU GFP-expressing S.tm (STG). 12 hours post infection, spleen, liver and serum samples were collected.

(A) Transcript levels of *Il1b*, *Il6* and *Tnf* genes normalized to *Gusb* mRNA levels determined by qRT-PCR

(B) Serum levels of IL1β, IL6, IL18 and TNFα measured by ELISA
(C) Bacterial burden of the spleen and liver determined by plating
Each point denotes single animal, bars with whiskers represent means ± SEM. Statistical significance was assessed with two-way ANOVA with Benjamini-Hochberg-corrected two-tailed post-hoc T tests (A) and Mann-Whitney U tests (B, C). In the plots, post-hoc test p values are indicated.

Supplementary Figure S7

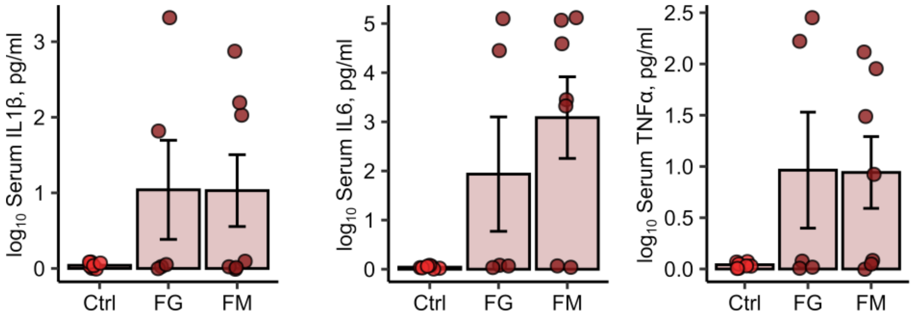

Administration of diverse intravenous iron preparations induces cytokine storm in *Fth*$^{\Delta/\Delta}$ animals upon S.tm infection.

LysM-Cre Fth$^{fl/fl}$ (*Fth*$^{\Delta/\Delta}$) mice mice were intravenously injected with PBS (n = 7) or 2 mg elementary Fe in form of iron gluconate (FG, n = 5) or iron isomaltoside (FM, n = 7) and infected three days later with 500 CFU GFP-expressing S.tm (STG). Animals were analyzed 20 hours post infection. Serum levels of IL1β, IL6 and TNFα were measured by ELISA.

Each point denotes single animal, bars represent with whiskers means ± SEM. Statistical significance was assessed with one-way ANOVA with Benjamini-Hochberg-corrected two-tailed post-hoc T tests. In the plots, post-hoc test p values are indicated.

Supplementary Figure S8

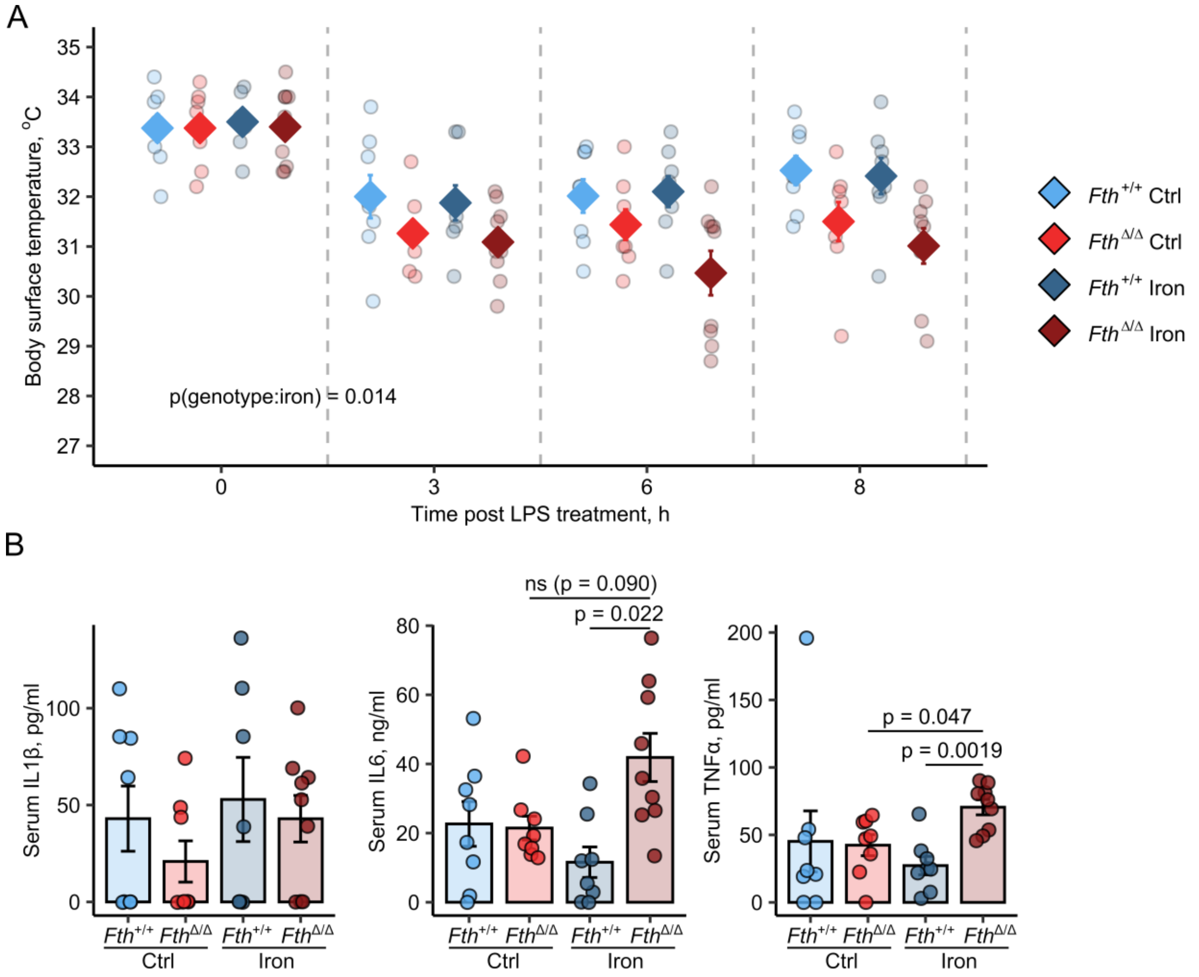

Iron loading induces LPS hyper-responsiveness in *Fth*$^{\Delta/\Delta}$ animals.

Fth$^{flox/flox}$ (*Fth*$^{+/+}$) and *LysM-Cre Fth*$^{fl/fl}$ (*Fth*$^{\Delta/\Delta}$) mice were intravenously administered PBS or iron isomaltoside (2 mg elementary Fe per animal) and intraperitoneally administered LPS (20 mg/kg) 3 days later (*Fth*$^{+/+}$ ctrl: n = 8, *Fth*$^{+/+}$ iron: n = 8, *Fth*$^{\Delta/\Delta}$ ctrl: n = 8, *Fth*$^{\Delta/\Delta}$ iron: n = 9). Mice were sacrificed 9 hours post LPS injection.

(A) Body surface temperature measured 0, 3, 6 and 8 hours post LPS injection.

(B) Serum levels of IL1β, IL6 and TNFα measured by ELISA.

In (A), each point represents a single animal, diamonds with whiskers represent means ± SEM. In other panels, each point denotes single animal, bars with whiskers represent means ± SEM. In (A), statistical significance was assessed with mixed-effect MANOVA (fixed effects: iron, genotype, time, genotype: iron interaction, genotype: time interaction, iron: time interaction, genotype: iron: time interaction, random effect: individual animal), significant p value for the genotype: iron interaction term is presented in the plot. In other panels, statistical significance was assessed with two-way ANOVA with Benjamini-Hochberg-corrected two-tailed post-hoc T tests. In the plots, post-hoc test p values are indicated.

Supplementary Figure S9

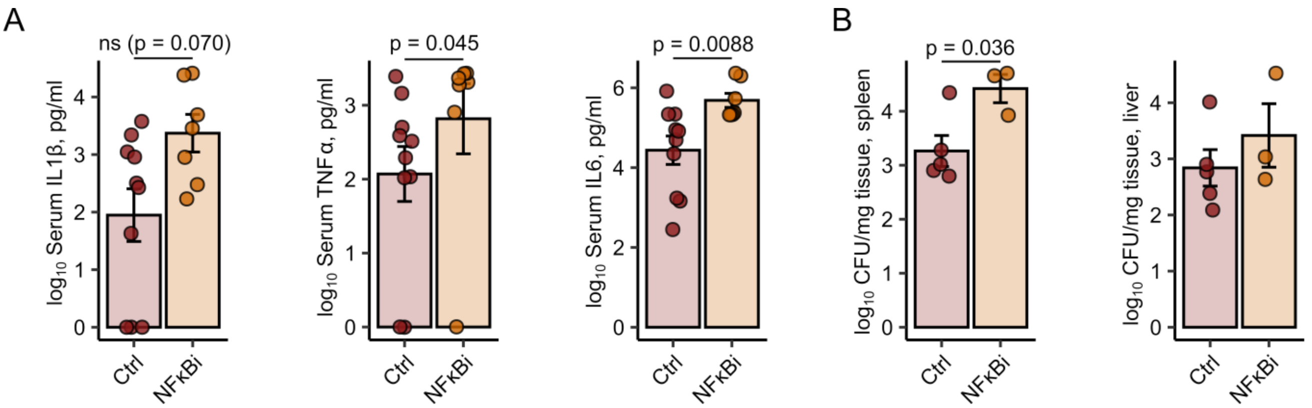

Administration of NFkB inhibitor in iron loaded *Fth*$^{\Delta/\Delta}$ animals leads to increased bacterial load and cannot reverse hyper-inflammation.

LysM-Cre Fth$^{fl/fl}$ (*Fth*$^{\Delta/\Delta}$) mice were intravenously administered iron isomaltoside (2 mg Fe) and infected three days later with 500 CFU GFP-expressing S.tm (Stg). Concomitantly with the infection mice were intraperitoneally co-injected with PBS (Ctrl, n = 11 for cytokins, n = 5 for CFU) or the NF-κB inhibitor BAY11-7082 (10 mg/kg, n = 7 for cytokines, n = 3 for CFU). Mice were analyzed 20 hours post infection.

(A) Serum levels of IL1β, IL6 and TNFα were measured by ELISA.

(B) Bacterial burden of the spleen and liver was determined by flow cytometry.

Bars with whiskers denote means ± SEM. Statistical significance was assessed with two-tailed T tests.

Supplementary Figure S10

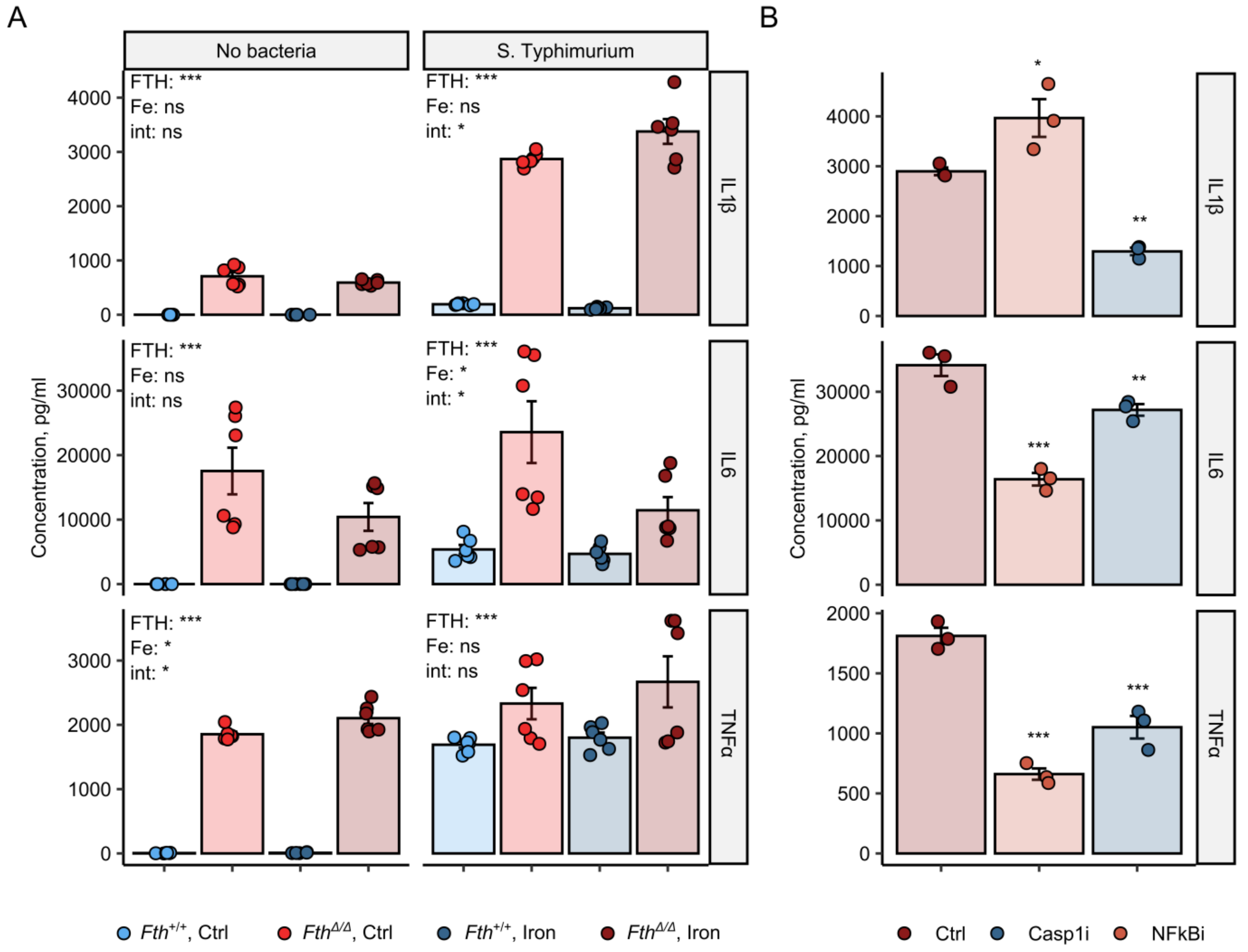

***Fth*$^{\Delta/\Delta}$ peritoneal macrophages produce IL1β, IL6 and TNFα in steady state with a dramatically increase upon infection with Salmonella.**

Peritoneal exudate macrophages (PEM) were isolated from *Fth*$^{flox/flox}$ (*Fth*$^{+/+}$) and *LysM-Cre Fth*$^{fl/fl}$ (*Fth*$^{\Delta/\Delta}$) mice and incubated for 3 hours with 50 µM Fe^{3+} ($FeCl_3$) or vehicle. Subsequently, cells were infected with MOI 10 S.tm or left untreated. In (B) cell were additionally stimulated with the NF-κB inhibitor BAY 11-7082 (NFkBi, 10 µg/ml), the caspase 1 inhibitor AC-YVAD-cmk (Casp1i, 1µM) or vehicle. Cell culture supranatants were harvested and analyzed 3 hours post infection.

(A) IL1β, IL6 and TNFα concentration in PEM culture supernatant 3 hours post infection measured by ELISA (n = 6 PEM pools, 3 – 5 animals each).

(B) IL1β, IL6 and TNFα concentration in supernatant of the PEM cultures treated with Caspase 1 or NF-κB inhibitor or vehicle determined by ELISA 3 hours post infection (n = 3 PEM pools, 3 – 5 animals each).

Bars with whiskers denote means ± SEM. In (A), statistical significance was assessed with two-way ANOVA separately for the control and infected cells with with Bonferroni post-hoc T tests; p values for ANOVA terms: genotype (FTH), iron (Fe) and their interaction (int) are presented in the plots. In (B), statistical significance was assessed with one way ANOVA with Bonferroni post-hoc T tests; post-hoc testing results for the control – treatment comparisons are presented in the plots. * $p < 0.05$, ** $p < 0.01$, *** $p < 0.001$.
